# A safeguarding factor monitors septal pore stability to secure compartmentalization during sporulation in *Bacillus subtilis*

**DOI:** 10.1038/s42003-026-10705-y

**Published:** 2026-07-15

**Authors:** Behzad Dehghani, Chris L. B. Graham, Helena Chan, Najwa Taib, Christopher D. A. Rodrigues

**Affiliations:** 1 School of Life Sciences, University of Warwick, Coventry, UK; 2 Australian Institute for Microbiology & Infection, University of Technology Sydney, Sydney, NSW Australia; 3 Hub Bioinformatics & Biostatistics, Department of Computational Biology, Institut Pasteur, Paris, France

**Keywords:** Cellular microbiology, Bacterial genetics

## Abstract

Bacterial endospore formation begins with a polar septum that compartmentalizes two transcriptionally distinct cells, the mother cell and forespore. In *Bacillus subtilis*, a complex composed of SpoIIIE, SpoIIIM and PbpG maintains compartmentalization at a septal pore during hydrolysis of septal peptidoglycan (PG), by coordinating two critical functions: chromosome translocation from the mother cell to the forespore through the septal pore and preservation of septal pore stability through SpoIIIE-PG interactions and PG synthesis. Disruption of this mechanism leads to cytoplasmic leakage, failed chromosome transfer, and reduced sporulation. Here, we identify CprV (YteV) as a safeguarding factor that maintains compartmentalization in response to septal pore stability defects. Cells lacking CprV show mild defects in compartmentalization and chromosome translocation; however, in mutants experiencing septal pore instability, loss of CprV significantly worsens these defects. Consistent with this role, CprV accumulates at the septal pore in a SpoIIIE-dependent manner when chromosome translocation is impaired. Computational analyses indicate that CprV exhibits structural similarity to Alba proteins found in Eukaryotes and Archaea and is primarily conserved in the *Bacillales*. Collectively, our data support a model whereby CprV monitors septal pore stability to safeguard compartmentalization during sporulation, providing insight into how cells monitor compartmental integrity during cellular remodeling.

**Figure:**
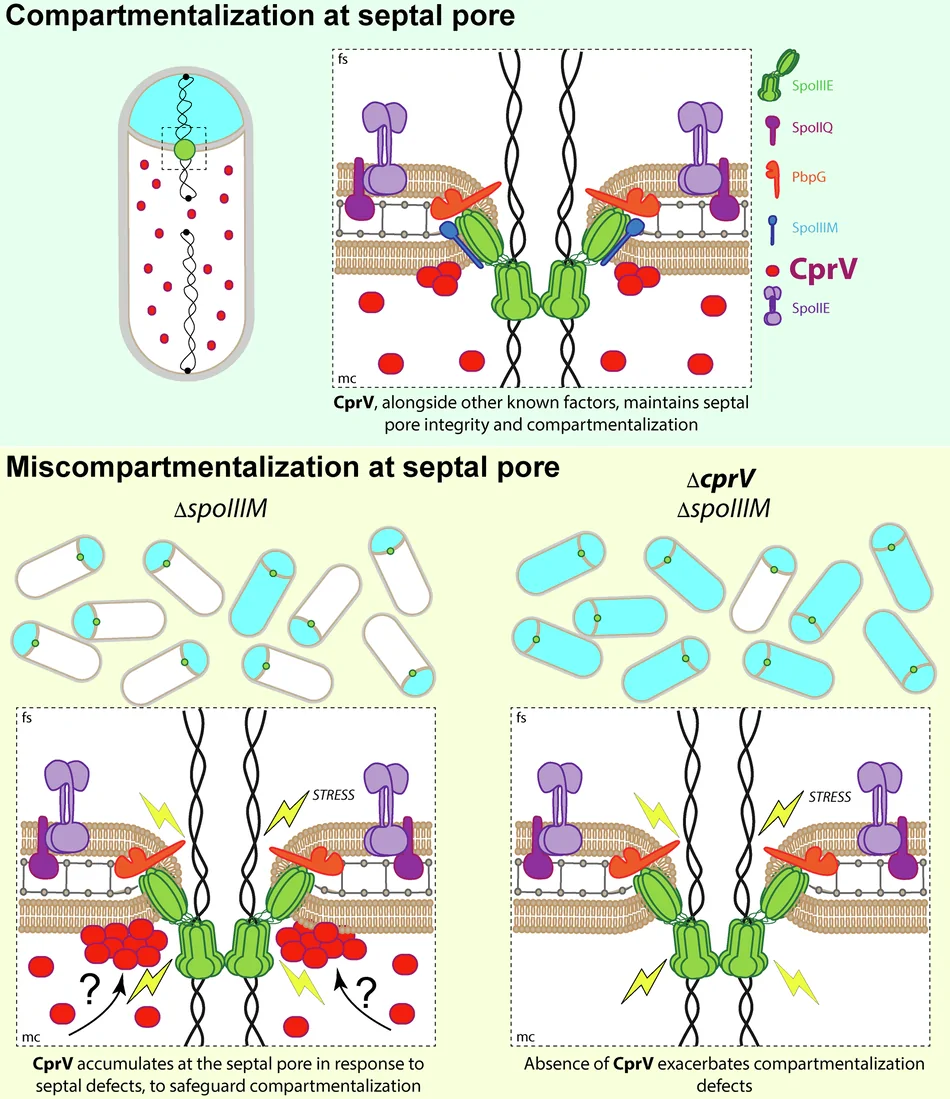

## Abstract (web summary)

Identification of CprV during bacterial sporulation reveals insight into how developing cells monitor compartmental integrity during cellular remodelling and differentiation.

## Introduction

Cellular differentiation underpins the development of specialized functions and depends on the establishment of distinct transcriptional programs within developing cells. During differentiation, maintenance of cellular compartmentalization ensures cells follow different developmental fates. In eukaryotes, cellular compartmentalization is mediated by membranes and, in some cases, reinforced by cell walls^1^. For example, during early embryogenesis, the formation of the blastocyst involves the segregation of the inner cell mass and the trophectoderm into distinct cellular compartments^2^. This compartmentalization, mediated by tight junctions and specialized membranes, ensures that each cell population activates different transcriptional programs leading to the development of embryonic tissues versus extraembryonic structures^3,4^. Despite its fundamental importance, the molecular mechanisms that ensure compartmentalization during differentiation remain poorly understood, even in evolutionarily ancient organisms, such as bacteria. Here, using the model organism *Bacillus subtilis*, we focus on an important example of how bacteria maintain compartmentalization during endospore formation (i.e., sporulation), a developmental process that leads to highly resistant dormant cells in several human, animal and insect pathogens, such as *Clostridioides difficile*, *Bacillus anthracis*, and *Paenibacillus larvae*.

**Fig. 1:**
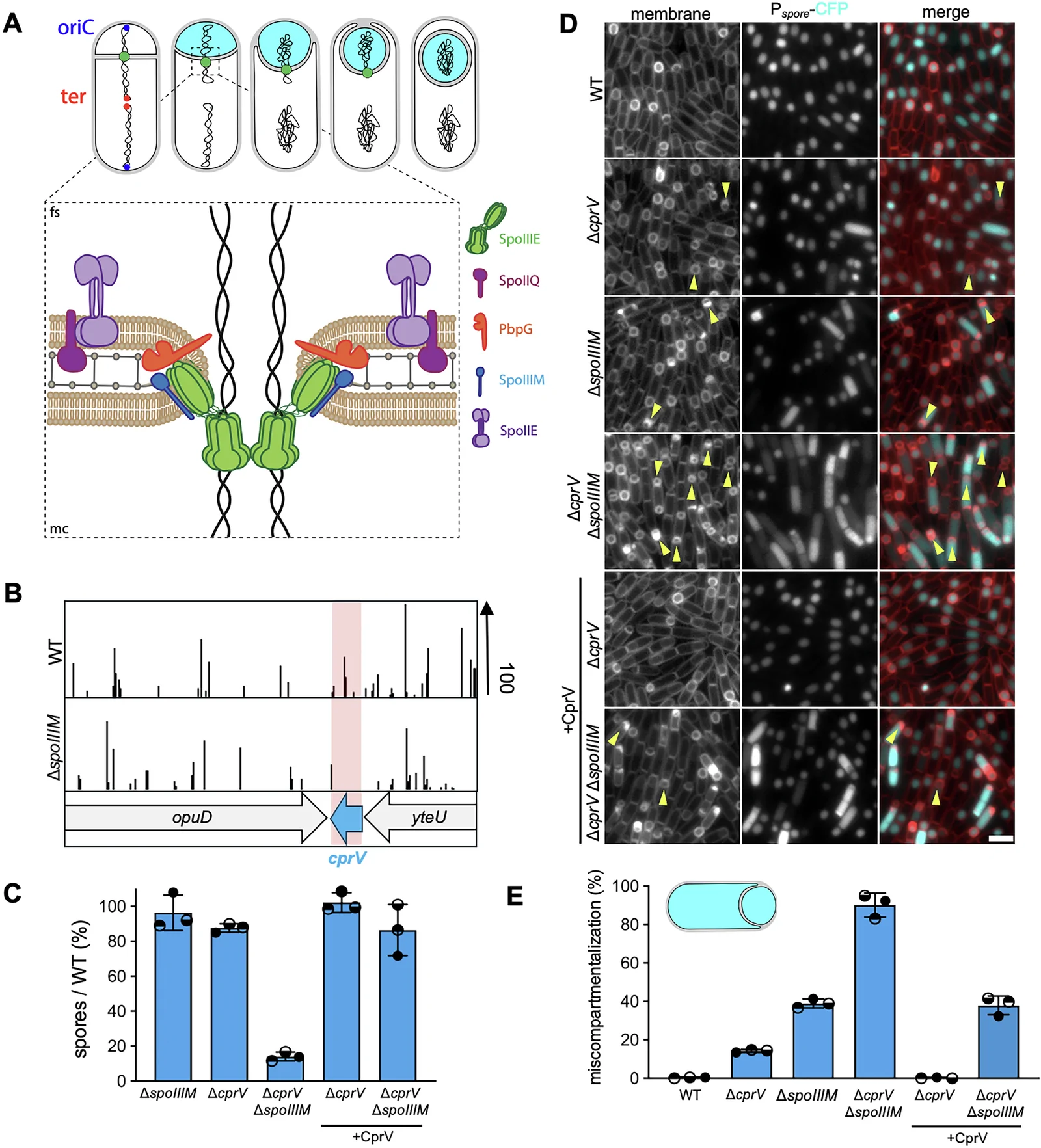
Identification of CprV and its requirement, together with SpoIIIM, in maintaining forespore morphology and compartmentalization. **A** Highly stabilized septal pore model. The septal pore is stabilized by SpoIIIE (green), PbpG (orange) and SpoIIIM (light blue). These factors coordinate chromosome translocation with PG remodeling during engulfment, while maintaining cytoplasmic and genetic compartmentalization. The septal pore is further stabilized by interactions between SpoIIE (light purple) and SpoIIQ (purple) (30). PG is shown as gray dots and lines. fs forespore, mc mother cell. **B** Tn-seq profiles in WT and Δ*spoIIIM* after 24 h of growth and sporulation in exhaustion medium. The height of each line reflects the number of sequencing reads at a given genomic position. Red box highlights the *cprV* (*yteV*) locus, which is depleted for transposon insertions in the Δ*spoIIIM* library compared to WT. The height of the arrow on the right indicates 100 transposon insertions. **C** Average sporulation efficiency (± SD, *n* = 3) of Δ*cprV*, Δ*spoIIIM*, and Δ*cprV* Δ*spoIIIM* and the respective *cprV* complementation strains. **D** Representative images of WT, ∆*cprV*, Δ*spoIIIM*, and Δ*cprV* Δ*spoIIIM* mutants and complementation strains expressing CFP (under the control of a σF-dependent promoter) at T3. Merged images show the membrane in red and CFP in cyan. Yellow arrows represent examples of forespore morphological defects in each background. Scale bar, 2 μm. **E** Average frequency (±SD of 3 biological replicates) of miscompartmentalized cells at T3 in WT, Δ*cprV*, Δ*spoIIIM*, and Δ*cprV* Δ*spoIIIM* (*n* > 150 per strain, per replicate).

One of the earliest events in sporulation is polar cell division, which generates two cellular compartments with distinct developmental fates (Fig. 1A). The smaller cell, called the forespore, matures into the dormant spore, whereas the larger mother cell nurtures spore development but eventually lyses to release the mature spore into the environment^5,6^. Each of these cells follows distinct developmental programs enforced by four sequentially acting, compartment-specific sigma factors (σ^F^, σ^E^, σ^G^, and σ^K^) that establish the divergent developmental fates of the forespore and mother cell^5,6^.

At the time of polar division, approximately 25% of the forespore chromosome is contained within the forespore, while the remaining 75% is contained within the mother cell^7^ (Fig. 1A). Translocation of the remainder of the forespore chromosome is achieved by a highly-conserved membrane-bound ATPase called SpoIIIE, which localizes as a single focus in the polar septum and translocates DNA into the forespore^8–10^. SpoIIIE is composed of three domains: an N-terminal transmembrane domain, a poorly conserved linker, and a motor domain^8,11–13^. The N-terminal transmembrane domain ensures SpoIIIE stability and localization to the center of the polar septum^8,14^. The SpoIIIE motor domain is subdivided into three subdomains (α, β, and γ) and is classified as a member of the RecA family of ATPases, sharing 78% sequence similarity to FtsK^11,12,15,16^. The motor assembles into a hexameric ring containing the ATPase and a channel that threads double-stranded DNA^12^. The SpoIIIE γ domain ensures directional DNA translocation by identifying chromosomal DNA sequence motifs designated SpoIIIE recognition sequences (SRSs)^17–20^.

Importantly, as the chromosome is translocated into the forespore, the peptidoglycan (PG) contained within the division septum undergoes remodeling by the balanced activity of hydrolytic and synthetic enzymes that drive a phagocytic-like process called engulfment^21,22^. During engulfment, the mother cell membranes migrate around the forespore, internalizing it within its cytoplasm^6,21^. Septal PG hydrolysis is essential for initiation for engulfment and depends on the DMP complex, containing SpoIID, SpoIIM, and SpoIIP^23–25^. This complex thins septal PG and creates space for the incorporation of newly synthesized PG that surrounds the forespore^22,26^. Initially, the DMP complex localizes near the center of the septum, but as engulfment begins it relocates to the leading edge of the migrating membranes^24,27^. Although several studies have identified a role for PG synthesis during engulfment, the exact identity of these PG synthases remains unclear^22,28,29^.

Recent work indicates that DNA translocation occurs through a small pore in the septal membrane, which forms because of polar septation over the partially segregated forespore chromosome^30^ (Fig. 1A). In addition, this work uncovered that DNA translocation is coordinated with septal PG remodeling at the onset of engulfment to ensure maintenance of mother cell and forespore compartmentalization at this septal pore^30^. Coordination is achieved through a complex composed of the aforementioned SpoIIIE, the bifunctional PG synthase PbpG (produced in the forespore) and a LysM-domain containing protein that is predicted to bind PG, called SpoIIIM (produced in the mother cell)^30^ (Fig. 1A). This complex preserves septal pore stability by maintaining SpoIIIE-PG interactions and by ensuring a balance between PG hydrolysis and synthesis around the pore at the onset of engulfment. This balance is critical to keeping the septal pore large enough to translocate DNA but small enough to prevent cytoplasmic leakage between these compartments (i.e., miscompartmentalization). Cells lacking SpoIIIE, PbpG, SpoIIIM, or both PbpG and SpoIIIM, exhibit varying degrees of septal pore instability that leads to septal pore enlargement, cytoplasmic miscompartmentalization and chromosome translocation defects^30^. Furthermore, in cells lacking any of these factors, and the highly conserved interaction between the phosphatase SpoIIE (which facilitates polar division and controls σ^F^ activation) and SpoIIQ in the forespore, the septal membrane retracts^30,31^. Importantly, blocking PG hydrolysis by deleting SpoIID and SpoIIP suppresses these phenotypes^30,31^. Together, this work led to a model whereby SpoIIIE, PbpG and SpoIIIM, and the SpoIIE–SpoIIQ interaction, counterbalance the destabilizing effect of PG hydrolysis around the septal pore, to maintain septal pore stability and ensure cytoplasmic and genetic compartmentalization.

Interestingly, while the absence of SpoIIIM and PbpG significantly affects compartmentalization, approximately 10% of cells remain compartmentalized and produce a similar number of viable spores^30^. This contrasts to the absence of SpoIIIE, where all cells are miscompartmentalized and no spores are formed^30,32,33^. These observations raised the possibility that additional SpoIIIE-dependent factors contribute to septal pore stability and compartmentalization. Here, we discover a role for CprV, formerly *yteV* or *cse60*^34^, in the coordination between PG remodeling and chromosome translocation, and maintenance of compartmentalization at the septal pore. Cells lacking CprV exhibit hallmark phenotypes associated with miscoordination of chromosome translocation and PG remodeling, and these phenotypes are significantly exacerbated in the absence of SpoIIIM and PbpG. Importantly, CprV is predominantly cytoplasmically localized in normal conditions, but in mutants experiencing compartmentalization or chromosome translocation defects, it becomes enriched at the septal membrane as a focus, in a SpoIIIE-dependent manner. However, CprV does not interact with SpoIIIE directly. Thus, CprV directly or indirectly senses a structural feature of the septal pore that requires SpoIIIE and is affected in mutants with septal pore stability defects. Given the toroidal architecture of the septal pore, delineated by membranes and PG, we propose CprV senses alterations to the architecture of the septal pore to safeguard compartmentalization in response to developmental defects that affect septal pore stability.

## Results

### A genetic and cytological screen identifies *cprV* as an additional compartmentalization factor

We previously showed that cells lacking SpoIIIM and PbpG exhibit miscompartmentalization in approximately 90% of sporulating cells and produce approximately 10% viable spores^30^. These data suggested that additional factors maintain compartmentalization and promote sporulation in the absence of SpoIIIM and PbpG, and could function to coordinate chromosome translocation with PG remodeling at the septal pore. To identify such factors, we used transposon-sequencing (Tn-seq) combined with fluorescence microscopy to screen for genes required for compartmentalization in the absence of *spoIIIM*, which results in miscompartmentalization in approximately 30–40% of cells^30^. Saturated transposon libraries were constructed in the WT and the Δ*spoIIIM* mutant strain (see “Methods”), and compartmentalization was monitored using a previously used forespore-produced fluorescent reporter protein (P_*spoIIQ*_-*cfp*)^30^. Analysis of the transposon insertion profiles revealed a set of genes in which insertions were underrepresented in the Δ*spoIIIM* mutant when compared to the WT (Figs. 1B and S1A). As expected, *pbpG* was on one of those genes (Fig. S1A). Deleting most of the other genes in combination with a Δ*spoIIIM* mutation did not increase miscompartmentalization (Fig. S1B). By contrast, combining Δ*yteV*, also known as *cse60*^34^ (renamed *cprV*; compartmentalization protein V), with the Δ*spoIIIM* mutant resulted in miscompartmentalization in the majority of cells, to a similar degree as observed in the Δ*spoIIIM* Δ*pbpG* (Fig. S1B).

CprV had been previously shown to be expressed in the mother cell under the control of σ^E^ and encodes a small, 60-amino-acid protein of unknown function^34^. To more carefully examine the role of CprV in compartmentalization and forespore morphology, we examined these phenotypes in the WT, Δ*crpV and* Δ*crpV* Δ*spoIIIM* mutant at 3-h after the onset of sporulation (T3), when most cells are approaching the final stages of engulfment and when compartmentalization and forespore morphological defects become more apparent in Δ*spoIIIM*^30^. While all WT sporangia were compartmentalized, in the Δ*cprV* and Δ*crpV* Δ*spoIIIM* double mutant, 14.3% and 90.1% of sporangia exhibited miscompartmentalization, respectively (Fig. 1D, E). Furthermore, while WT forespores had a normal oblong appearance, Δ*cprV* displayed abnormal looking forespores in a minor proportion of the population, including smaller forespores or forespores with irregular membranes (Fig. 1D and S1C). These forespore morphological defects became more predominant in the Δ*crpV* Δ*spoIIIM* double mutant (Fig. 1D and S1C). In further validation of the Δ*spoIIIM* Tn-seq screen data, the Δ*cprV* Δ*spoIIIM* double mutant produced 6.5-fold fewer heat-resistant spores (14.1%) relative to Δ*cprV* (90.6%) (Fig. 1C). Ectopic expression of *cprV* at the *yhdG* locus restored sporulation and compartmentalization in both Δ*cprV* and Δ*cprV* Δ*spoIIIM* mutants to near wild-type and Δ*spoIIIM* mutant levels, respectively (Fig. 1C, E). Collectively, these data identify CprV as an additional factor required for compartmentalization and suggest it functions with SpoIIIM in maintaining compartmentalization during sporulation.

### CprV is required for septal pore stability

To begin to understand CprV’s role in compartmentalization and whether it functions in the same pathway as SpoIIIM, we tested if CprV contributes to stabilization of the septal pore. Previous work suggests SpoIIIM contributes to stabilization of the septal pore by counterbalancing the effect of the engulfment hydrolases, SpoIID and SpoIIP, which thin septal PG in the polar septum^21,23,24^. Specifically, it was shown that the absence of SpoIID and SpoIIP, which results in the polar septum remaining flat, suppresses the compartmentalization defects of the Δ*spoIIIM* and Δ*spoIIIM* Δ*pbpG* mutants^30^. Thus, we wanted to know if the absence of SpoIID and SpoIIP would suppress the Δ*cprV* and Δ*cprV* Δ*spoIIIM* compartmentalization defects. Deleting *spoIID* and *spoIIP* suppressed the compartmentalization defect of Δ*cprV* and Δ*cprV* Δ*spoIIIM* (Fig. S2A). This suggests that CprV, like SpoIIIM, maintains compartmentalization by counterbalancing the effects of PG hydrolysis around the septal pore.

To further explore the role of CprV in septal pore stabilization and its genetic relationship with SpoIIIM, we took advantage of another phenotype associated with the absence of SpoIIIM. Previous research showed that the absence of SpoIIIM and the forespore protein SpoIIQ leads to increased septal pore instability, resulting in septal PG being completely digested by SpoIIP and SpoIID and subsequent dissolution of the polar septum at the onset of engulfment^30,31^. This phenotype is called septal retraction and results in miscompartmentalized sporangia that lack a visible polar septum; instead, vestiges of the septa are observed^30,31^. To investigate whether the absence of CprV in Δ*spoIIQ* cells causes septal retraction, we examined the Δ*spoIIQ* Δ*cprV* double and Δ*spoIIQ* Δ*spoIIIM* Δ*cprV* triple mutants for signs of septal retraction. Septal retraction was apparent in both mutants, and quantification showed that 19.7% of Δ*spoIIQ* Δ*cprV* and 90.3% of Δ*spoIIQ* Δ*spoIIIM* Δ*cprV* sporangia exhibit this phenotype (Fig. S3A, B). For comparison, we also re-examined septal retraction in the Δ*spoIIQ* Δ*spoIIIM* double mutant, which resulted in 59.2% retracted septa, similar to published data (Fig. S3A, B)^30,31^. Thus, CprV is also required for septal stability, although to a lesser extent than SpoIIIM. Finally, as septal retraction in Δ*spoIIQ* Δ*spoIIIM* depends on the SpoIID and SpoIIP activity, we wanted to know if this was also the case in the Δ*spoIIQ* Δ*cprV* and Δ*spoIIQ* Δ*spoIIIM* Δ*cprV* double and triple mutants, respectively. Indeed, deleting *spoIID* and *spoIIP* completely suppressed septal retraction, and compartmentalization was restored in both strains (Fig. S2B). These findings indicate that CprV, like SpoIIIM, contributes to septal pore stability during engulfment.

### CprV is required for the dispersal of SpoIIIE complexes

**Fig. 2:**
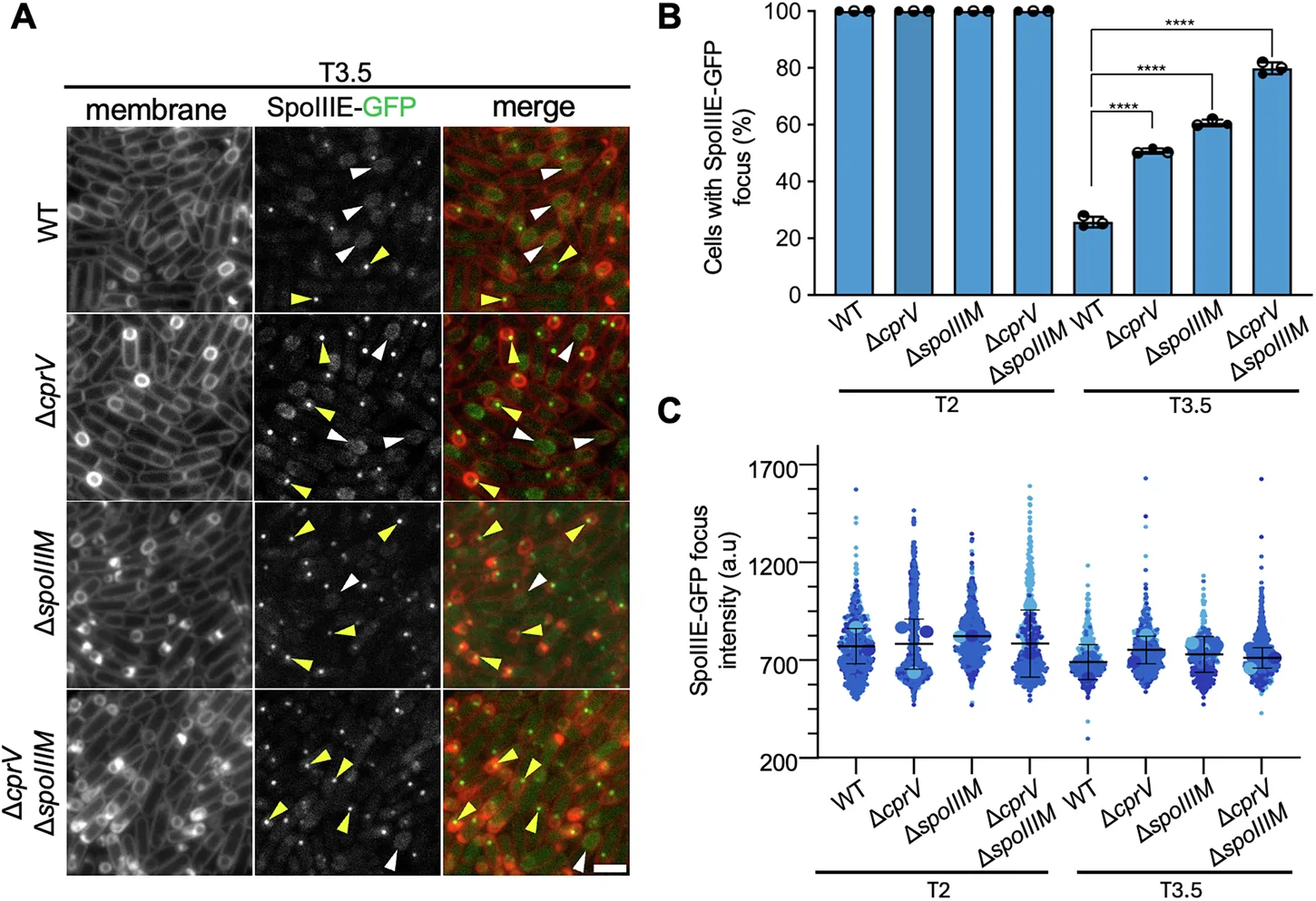
CprV is required for the dispersal of SpoIIIE complexes. **A** Representative images of SpoIIIE-GFP localization in WT, Δ*cprV*, Δ*spoIIIM*, and Δ*cprV* Δ*spoIIIM* cells at T3.5. Scale bar, 2 μm. Yellow arrows point to the examples of SpoIIIE-GFP focus, and white arrows point to SpoIIIE-GFP dispersal. **B** Average frequency (±SD of 3 biological replicates) of cells with SpoIIIE-GFP foci in WT, Δ*cprV*, Δ*spoIIIM* and Δ*cprV* Δ*spoIIIM* mutants at T2 and T3.5 (*n* > 250 per time-point per strain, per replicate). **C** Average fluorescence intensity of SpoIIIE-GFP foci (±STDEVP, *n* > 200, per time-point, per strain) in WT, Δ*cprV*, Δ*spoIIIM* and Δ*cprV* Δ*spoIIIM* mutants during at T2 and T3.5.

One way in which CprV could contribute to septal pore stability and compartmentalization is by ensuring efficient SpoIIIE localization at the septal pore, which is absolutely required for compartmentalization. Although the absence of SpoIIIM does not impact SpoIIIE localization^30^, we considered the possibility that the absence of both CprV and SpoIIIM could lead to SpoIIIE becoming mislocalized and therefore underlie the severe miscompartmentalization of the Δ*cprV* Δ*spoIIIM* mutant. To test this idea, we examined SpoIIIE-GFP localization in WT and Δ*cprV* Δ*spoIIIM* double mutant cells at 2 (T2) (Fig. S3C) and 3.5 h (T3.5) after the onset of sporulation (Fig. 2A), to coincide with active engulfment and engulfment completion in most sporangia. Consistent with previous reports^8,30,35,36^, in WT cells SpoIIIE-GFP localizes as a discrete focus at or near the center of the curving septal membrane and as cells progress through engulfment the focus is often located in the engulfing membranes (Fig. S3C). As engulfment nears completion, the focus disperses, with SpoIIIE-GFP localizing as a diffuse signal in the forespore membranes of most cells^30^ (Fig. 2A). SpoIIIE-GFP dispersal is thought to represent detachment of SpoIIIE from the DNA^37^. In Δ*cprV* Δ*spoIIIM* double mutant sporangia, SpoIIIE-GFP localized as a single focus in the engulfing membrane (T2) (Fig. S3C). However, in sporangia that had completed engulfment, SpoIIIE-GFP retained its localization as a focus in a high proportion of cells (Fig. 2A). These data are reminiscent of what has been reported in the Δ*pbpG* Δ*spoIIIM* double mutant^30^. Quantification of the number of cells with a SpoIIIE-GFP focus at T2 and T3.5 confirmed these observations (Fig. 2B). In the Δ*crpV* mutant we also observed a higher frequency of cells with SpoIIIE-GFP foci at T3.5 (50.8%), relative to the WT strain (25.8%) (Fig. 2B). Furthermore, quantification of SpoIIIE-GFP foci intensity indicates that SpoIIIE-GFP signal intensity is similar between WT, Δ*cprV*, Δ*spoIIIM* and Δ*cprV* Δ*spoIIIM* double mutant cells (Fig. 2C). These data indicate that miscompartmentalization in the Δ*cprV* Δ*spoIIIM* double mutant is not due to mislocalization of SpoIIIE and that CprV, like SpoIIIM, contributes to dispersal of SpoIIIE complexes.

### CprV is required for efficient chromosome translocation and chromosome retention in the forespore

Our data thus far point to the possibility that CprV’s role in septal pore stability and compartmentalization is closely related to SpoIIIM, which interacts with SpoIIIE and ensures compartmentalization by coordinating chromosome translocation with PG remodeling at the septal pore^30^. In cells lacking SpoIIIM, this coordination mechanism is compromised, and chromosome translocation defects occur in 10–15% of sporangia^30^. Specifically, a subset of Δ*spoIIIM* mutant sporangia initially fail to translocate the chromosome into the forespore, and then the entire chromosome is effluxed back into the mother cell^30^. Importantly, these chromosome translocation defects are suppressed when septal PG hydrolysis is blocked by deleting *spoIID* and *spoIIP*^30^. If CprV functions with SpoIIIM in this coordination mechanism, it may also be required for efficient chromosome translocation, and cells lacking both CprV and SpoIIIM may exhibit increased chromosome translocation defects.

**Fig. 3:**
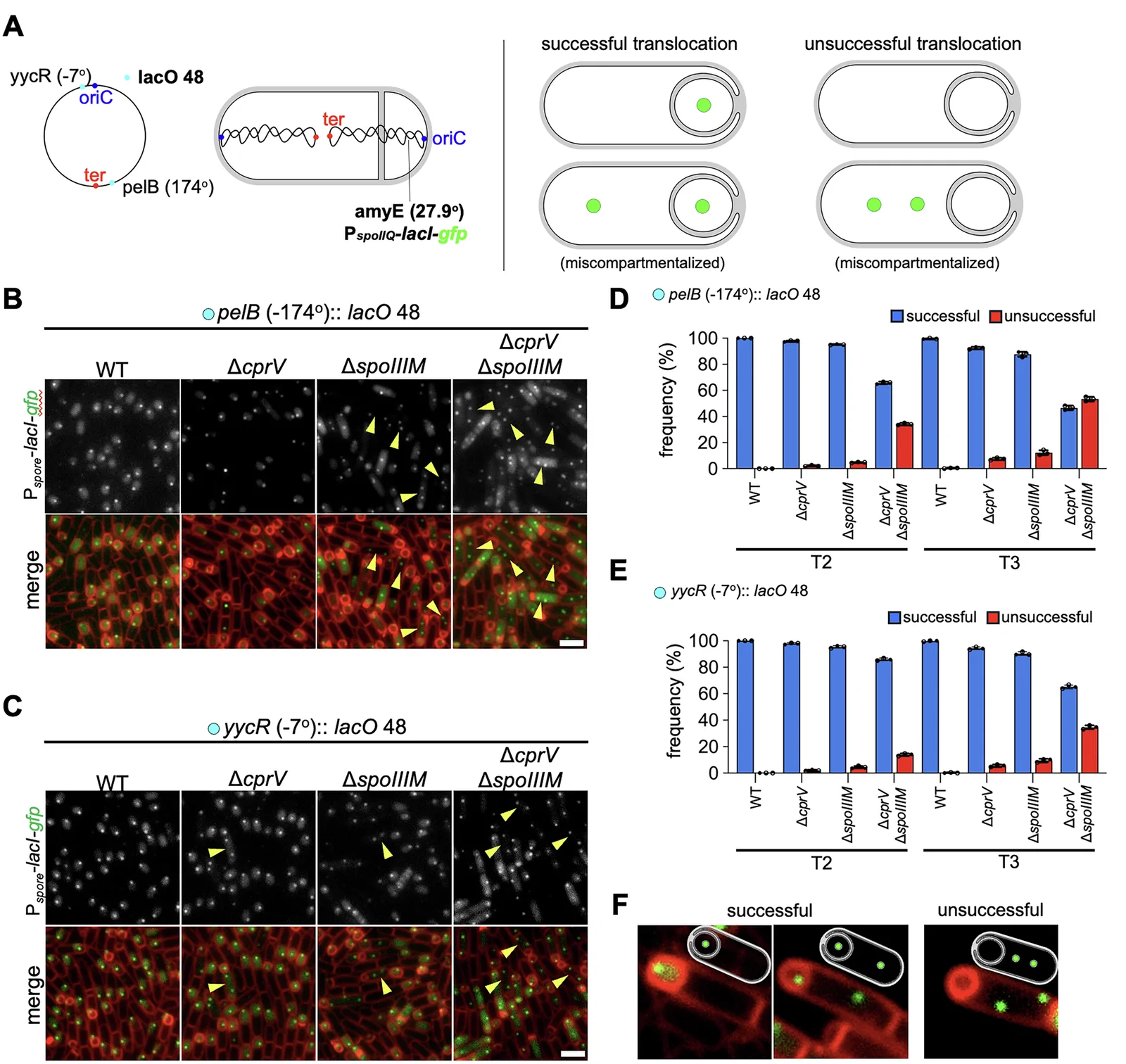
CprV and SpoIIIM are required for efficient chromosome translocation and forespore chromosome retention. **A** Diagram illustrating the rationale of the LacI–*lacO* system used to visualize chromosome translocation and chromosome retention in the forespore. LacI-GFP, expressed in the forespore from the P_*spoIIQ*_ promoter at the *amyE* locus, binds to *lacO48* arrays inserted at the *pelB* or *yycR* chromosomal loci. The chromosome origin (oriC) and terminus (ter) are shown in blue and red, respectively. Successful chromosome translocation (or chromosome origin retention) is observed by a single LacI-GFP focus in the forespore (and an additional focus in the mother cell if miscompartmentalization occurs). Unsuccessful translocation is indicated by the absence of a LacI-GFP focus in the forespore (or by two foci in the mother-cell in miscompartmentalized cells). **B** Representative images of LacI-GFP foci at T3 in WT, Δ*cprV*, Δ*spoIIIM*, and Δ*cprV* Δ*spoIIIM* strains carrying *lacO48* at the *pelB* locus. Yellow arrows represent examples of unsuccessful chromosome translocation in each background. Scale bar, 2 μm. **C** Representative images of LacI-GFP foci at T3 in WT, Δ*cprV*, Δ*spoIIIM*, and Δ*cprV* Δ*spoIIIM* strains carrying *lacO48* at the *yycR* locus. Yellow arrows represent examples of unsuccessful chromosome retention in each background. Scale bar, 2 μm. **D** Average frequency (±SD, 3 biological replicates) of cells displaying successful translocation or unsuccessful translocation at T2 and T3, with *lacO48* integrated at *pelB* in WT, Δ*cprV*, Δ*spoIIIM*, and Δ*cprV* Δ*spoIIIM* strains. **E** Average frequency (±SD, 3 biological replicates) of cells displaying successful translocation or unsuccessful translocation at T2 and T3, with *lacO48* integrated at *yycR* in WT, Δ*cprV*, Δ*spoIIIM*, and Δ*cprV* Δ*spoIIIM* strains (*n* > 150 per strain, per replicate, per time point). **F** Representative images of successful and unsuccessful chromosome translocation with *lacO48* at *pelB*. Successful translocation: LacI-GFP focus in forespore; unsuccessful translocation: two mother-cell LacI-GFP foci.

To test this, we employed the LacI-lacO system utilized previously^30^. Strains were engineered to express LacI-GFP under the control of a forespore-specific promoter (P_*spoIIQ*_-*lacI*-*gfp*), integrated at the *amyE* locus, present in the forespore following polar division^7^. Additionally, these strains were modified to include 48 *lacO* repeats either at the *pelB* locus, near the terminus, or at *yycR* locus, near the origin of replication (Fig. 3A). In strains with *lacO* inserted at the *pelB* locus, a LacI-GFP focus appears in the forespore only if the terminus region of the chromosome is successfully translocated (Fig. 3A, F). Conversely, with *lacO* inserted at *yycR*, a LacI-GFP focus is observed in the forespore unless that region of the chromosome is effluxed back into the mother cell^30^. Thus, strains with *lacO* repeats at *pelB* allow monitoring of successful chromosome translocation, while those with *lacO* repeats at *yycR* verify successful retention of the origin region of chromosome in the forespore. Furthermore, in sporangia exhibiting miscompartmentalization, if there is unsuccessful translocation of the terminus region chromosome, or failure to retain the origin region in the forespore, two LacI-GFP foci are observed in the mother cell (Fig. 3A, F). Importantly, since chromosome translocation takes approximately 20 min^38,39^, and most WT sporangia have translocated the chromosome by T2^30^, by comparing chromosome translocation frequency at the *pelB* locus at two different points, T2 and T3, we can deduce whether the terminus region of the chromosome remains in the forespore after it has been translocated or not.

At T2 and T3 in strains with *lacO* near the terminus (*pelB*), all WT sporangia translocated the chromosome into the forespore (Figs. 3D and S3D). By contrast, in the absence of CprV, 2.2% of sporangia failed to translocate into the forespore at T2 (Figs. 3D and S3D), and this increased to 7.5% at T3 (Fig. 3B, D). By contrast, in the Δ*cprV* Δ*spoIIIM* double mutants, 34.2% of cells failed to complete chromosome translocation at T2 (Figs. 3D and S3D), with this proportion rising to 53.4% by T3 (Fig. 3B, D).

A similar trend was observed in strains with *lacO* repeats near the origin (*yycR*): in Δ*cprV* cells, 2.1% of the forespores failed to retain the origin region in the chromosome at T2 (Figs. 3E and S3E), and this increased to 5.7% at T3 (Fig. 3C, E). By contrast, in the Δ*cprV* Δ*spoIIIM* double mutant there was an increase in origin retention defects, from 13.9% at T2 (Figs. 3E and S3E) to 34.6% at T3 (Fig. 3C, E). Importantly, deleting *spoIID* and *spoIIP* suppressed these chromosome translocation defects, and all sporangia successfully translocated the chromosome into the forespore (examination of *lacO* at *pelB;* Fig. S2C).

Taken together, these findings suggest that like SpoIIIM, CprV is required for efficient chromosome translocation and chromosome retention in the forespore and that the absence of both proteins increases these defects. Thus, CprV functions with SpoIIIM in promoting efficient chromosome translocation and chromosome retention in the spore.

### *cprV* exhibits a synthetic genetic relationship with *pbpG*

To further test if CprV’s role in maintaining compartmentalization is connected to coordination between chromosome translocation and PG remodeling at the septal pore, we asked whether *cprV* exhibits a synthetic genetic interaction with *pbpG*, which interacts with SpoIIIE and contributes to this coordination mechanism^30^. To this end, we examined the phenotypes reported thus far (sporulation efficiency, miscompartmentalization, septal stability, and chromosome translocation) in the Δ*cprV* Δ*pbpG* and Δ*pbpG* mutants (Fig. S4).

In terms of sporulation efficacy, we observed a significant reduction in the frequency of heat-resistant spores in the Δ*cprV* Δ*pbpG* double mutant (21.1%) relative to Δ*pbpG* (89.1%) (Fig. S4A). In terms of compartmentalization and spore morphology, miscompartmentalization increased from 6% in Δ*pbpG* to 98.5% in the Δ*cprV* Δ*pbpG* double mutant (Fig. S4B, D), which was accompanied by morphological defects in the majority of forespores, including reduced forespore size and irregularly shaped forespore membranes. (Fig. S4B). Examination of septal retraction in the Δ*cprV* Δ*pbpG* Δ*spoIIQ* background showed an increase to 90.1%, compared with 56.2% in Δ*pbpG* Δ*spoIIQ* (Fig. S4C, D). Evaluation of chromosome translocation in the Δ*cprV* Δ*pbpG* double mutant using both the *pelB* and *yycR* loci showed that the double mutant is more severely impacted in chromosome translocation and chromosome retention than the Δ*pbpG* mutant (Fig. S4E–G). Specifically, at T2 and T3, the frequency of sporangia in the Δ*cprV* Δ*pbpG* double mutant that had failed to translocate the terminus region (*pelB locus)* into the forespore increased from 9.8 to 50.1% (Fig. S4E). Similarly, the frequency of sporangia in the Δ*cprV* Δ*pbpG* double mutant that failed to retain the origin region in the forespore (*yycR* locus) increased from 1.5 to 37.5% (Fig. S4F). These data suggest that CprV is required for chromosome translocation, and its retention in the forespore, in the absence of PbpG. Finally, while deleting *spoIID* and *spoIIP* suppressed the translocation defects in virtually all sporangia of the Δ*cprV* Δ*pbpG* double mutant (examination of *lacO* at *pelB;* Fig. S2C), 26% of cells remained miscompartmentalized (Fig. S2A). Thus, abolishing septal PG hydrolysis is not sufficient to promote compartmentalization in all cells, in the absence of CprV and PbpG.

Together, these data identify a synthetic genetic interaction between *cprV* and *pbpG* and suggest that CprV, SpoIIIM, and PbpG together ensure efficient coordination between chromosome translocation and PG remodeling to maintain compartmentalization at the septal pore.

### Sporangia lacking CprV, PbpG, and SpoIIIM are severely defective in coordinating chromosome translocation with PG remodeling

**Fig. 4:**
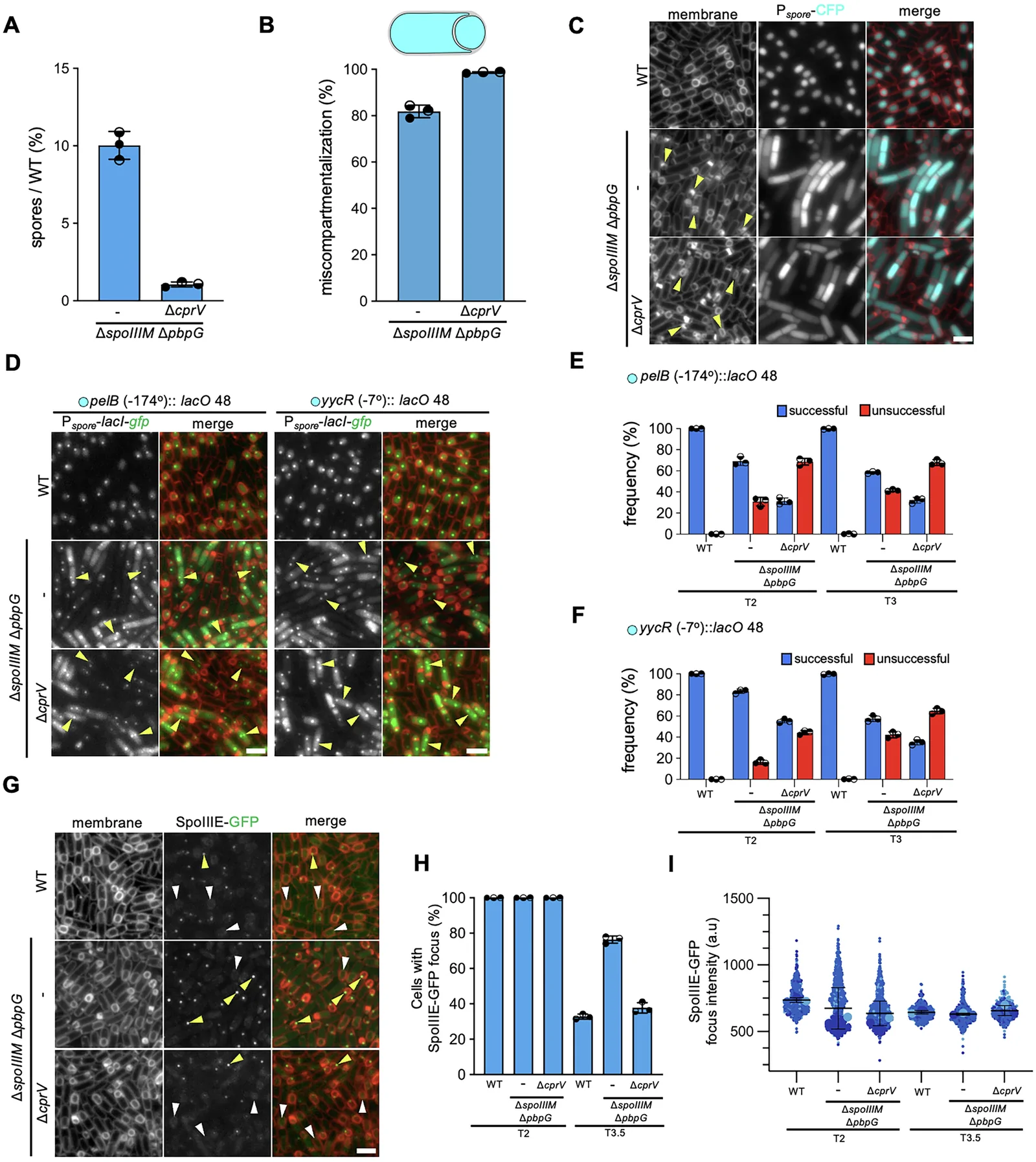
The triple mutant lacking CprV, SpoIIIM, and PbpG exhibits severe defects in septal integrity, chromosome translocation, and SpoIIIE localization dynamics. **A** Average sporulation efficiency (% ± SD, *n* = 3) in Δ*spoIIIM* Δ*pbpG *double mutant and Δ*cprV* Δ*spoIIIM* Δ*pbpG* triple mutant. **B** Average frequency (±SD of 3 biological replicates) of miscompartmentalized cells at T3 in Δ*spoIIIM* Δ*pbpG* double mutant and Δ*cprV* Δ*spoIIIM* Δ*pbpG* triple mutant (*n* > 250 per time-course, per strain, per replicate). **C** Representative images of miscompartmentalization in WT, Δ*spoIIIM* Δ*pbpG*, and Δ*cprV* Δ*spoIIIM* Δ*pbpG* triple mutant at T3. **D** Representative images of LacI-GFP foci at T3 in WT, Δ*spoIIIM* Δ*pbpG*, and Δ*cprV* Δ*spoIIIM* Δ*pbpG* triple mutant strains containing *lacO48* inserted at *pelB* locus and *yycR* locus. Yellow arrows represent examples of unsuccessful chromosome translocation (or chromosome retention) in each background. Scale bar, 2 μm. **E** Average frequency (±SD of 3 biological replicates) of cells exhibiting successful or unsuccessful chromosome translocation at T2 and T3, with *lacO48* integrated at the *pelB* locus (174°) in WT, Δ*spoIIIM* Δ*pbpG*, and Δ*cprV* Δ*spoIIIM* Δ*pbpG* triple mutant (*n* > 250 per time-course, per strain, per replicate). **F** Average frequency (±SD of 3 biological replicates) of cells exhibiting successful or unsuccessful chromosome translocation at T2 and T3, with *lacO48* integrated at the *yycR* locus in WT, Δ*spoIIIM* Δ*pbpG* and Δ*cprV* Δ*spoIIIM* Δ*pbpG* triple mutant (*n* > 250 per time-course, per strain, per replicate). **G** Representative images of SpoIIIE-GFP localization in WT, Δ*spoIIIM* Δ*pbpG* and Δ*cprV* Δ*spoIIIM* Δ*pbpG* triple mutant at T3.5. Yellow arrows point to SpoIIIE-GFP foci and white arrows to SpoIIIE-GFP dispersal. Scale bar, 2 μm. **H** Average frequency (±SD of 3 biological replicates) of cells with a SpoIIIE-GFP in WT, Δ*spoIIIM* Δ*pbpG* and Δ*cprV* Δ*spoIIIM* Δ*pbpG* triple mutant at T2 and T3.5 (*n* > 200 per time-course, per strain, per replicate). **I** Average fluorescence (±STDEVP, *n* > 200, per time-point, per strain) intensity of SpoIIIE-GFP foci in WT, Δ*spoIIIM* Δ*pbpG* and Δ*cprV* Δ*spoIIIM* Δ*pbpG* triple mutant at T2 and T3.5.

Our data thus far suggest that coordination between chromosome translocation and PG remodeling at the septal pore to maintain compartmentalization, requires CprV, SpoIIIM and PbpG. We wanted to know the effect of deleting all three proteins on these processes and specifically examine a role for CprV in the Δ*spoIIIM* Δ*pbpG* double mutant, which despite exhibiting severe defects is still able to sustain sporulation in 10% of the population^30^. To do so, we compared sporulation efficiency, miscompartmentalization and chromosome translocation in the Δ*cprV* Δ*spoIIIM* Δ*pbpG* triple mutant strains to the Δ*spoIIIM* Δ*pbpG* double mutant (Fig. 4).

The triple mutant exhibited a 10-fold reduction in sporulation efficiency relative to the double mutant, with only 1.1% heat-resistant spores in the triple mutant (Fig. 4A). Miscompartmentalization increased to 98.7% in the triple mutant, compared to the 81.9% in the double mutant (Fig. 4B, C). In addition, in the triple mutant, virtually all forespores displayed morphological defects (Fig. 4C). Similarly, chromosome translocation and chromosome retention defects were exacerbated in the triple mutant relative to the double mutant (Fig. 4D–F). Specifically, at T2, the triple mutant resulted in a two-fold increase in terminus (*pelB*) translocation defects relative to the double mutant (Fig. 4D, E), with 67.8% of sporangia failing to translocate the chromosome into the forespore in the triple mutant. Similarly, at T3, there was a 1.5-fold increase in the number of forespores with origin (*yycR* locus) retention defects relative to the double mutant, with 64.7% of sporangia failing to retain the origin in the forespore in the triple mutant (Fig. 4D, F). Thus, CprV plays an important role in ensuring efficient chromosome translocation and chromosome retention in the absence of SpoIIIM and PbpG.

Interestingly, looking at SpoIIIE-GFP localization, while the triple mutant did not compromise SpoIIIE-GFP focal intensity at T2 or T3.5 (Figs. 4G, I, and S3C), the frequency of SpoIIIE-GFP focal localization at T3.5 was lower in the triple mutant compared to the double mutant, the latter of which remained high compared to WT (Fig. 4G, H), as previously reported^30^. Since SpoIIIE-GFP focus formation is thought to represent attachment to DNA^37^, these observations suggest that removal of CprV in cells lacking SpoIIIM and PbpG results in SpoIIIE detachment from the DNA. As the triple mutant exhibits a higher frequency of chromosome translocation and chromosome retention defects than the double mutant (Fig. 4E, F), it is likely that fewer forespores retain DNA at the septal pore, which in turn would result in SpoIIIE focus disassembly to levels that are coincidently similar to WT (Fig. 4H).

Finally, while deleting *spoIID* and *spoIIP* suppressed the chromosome translocation defect of the double and triple mutant (examination of *pelB* locus; Fig. S2C), a fraction of cells remained miscompartmentalized in the triple mutant (33%), but not the double mutant (Fig. S2A). Thus, abolishing septal PG hydrolysis is not sufficient to restore compartmentalization in the absence of all three proteins.

Together, these data support a model whereby CprV, SpoIIIM and PbpG together are required for the coordination between chromosome translocation and PG remodeling, which maintains compartmentalization at the septal pore. These data also suggest that CprV plays an important role in facilitating chromosome translocation in cells lacking SpoIIIM and PbpG, and points to a more direct involvement of CprV in chromosome translocation.

### CprV assembles into a SpoIIIE-dependent septal focus that increases in frequency in response to chromosome translocation defects

**Fig. 5:**
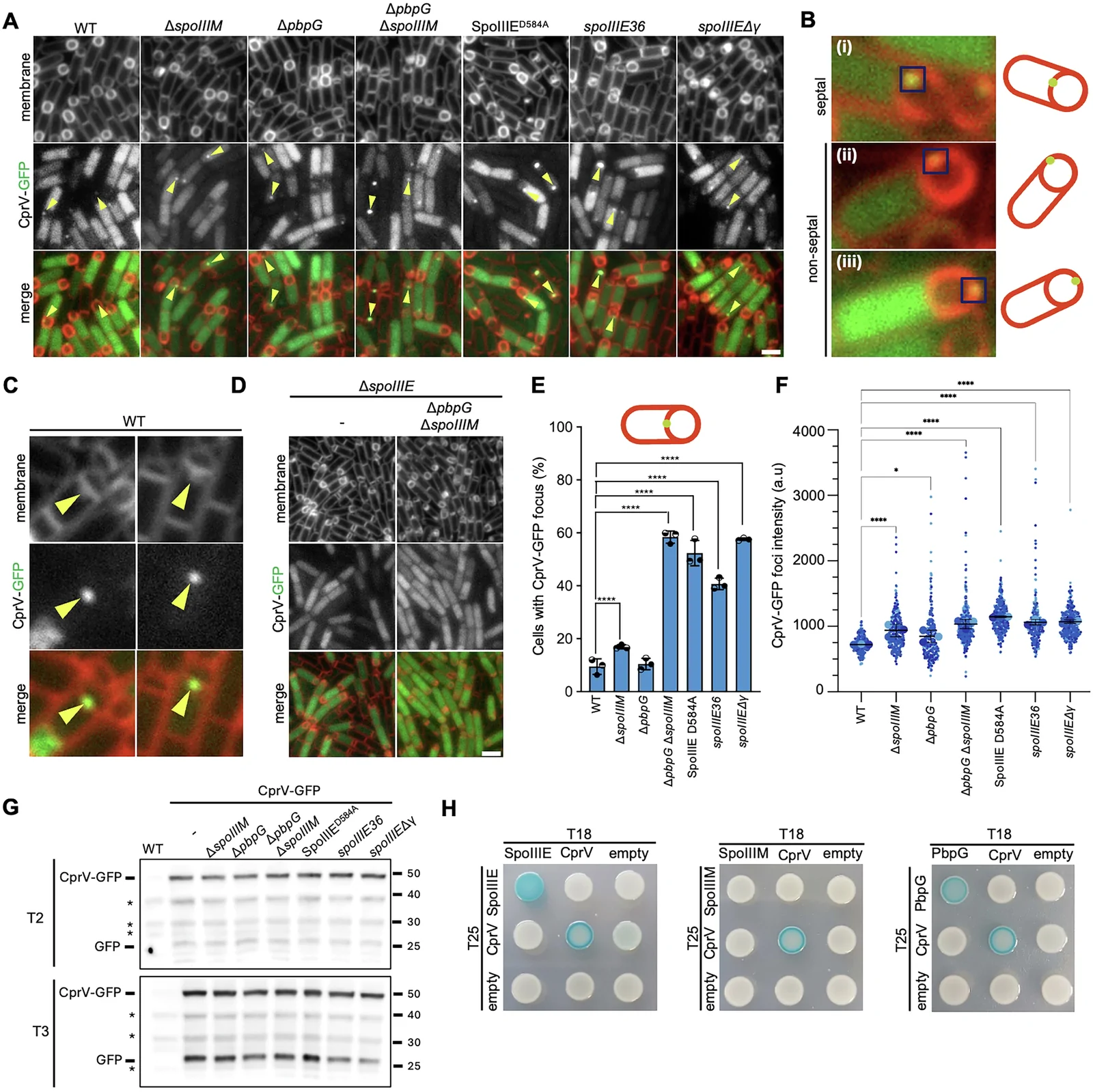
CprV recruitment to the septal pore depends on SpoIIIE and is enhanced in mutants with compartmentalization and chromosome translocation defects. **A** Representative images showing CprV–GFP localization in WT, Δ*spoIIIM*, Δ*pbpG*, Δ*pbpG* Δ*spoIIIM*, SpoIIIE^D584A^, *spoIIIE36* and *spoIIIEΔγ* strains at T2. Yellow arrowheads indicate CprV–GFP foci. **B** Different spatial positions of CprV-GFP are observed along the engulfing membrane in WT cells; septal membrane (i); lateral engulfing membrane (ii) and forespore pole (iii). **C** Enlarged representative images showing increased septal localization of CprV-GFP in WT cells with septal defects. **D** Representative images of CprV-GFP localization in Δ*spoIIIE* and Δ*pbpG ΔspoIIIM ΔspoIIIE* mutants. In the absence of *spoIIIE*, CprV-GFP foci are not present. **E** Frequency (mean ± SD from three biological replicates) of cells containing a CprV-GFP septal focus in the indicated strains at T2 (*n* > 200 cells per time point, per strain, per replicate); ****p < 0.0001, Mann–Whitney test. **F** Quantification of CprV–GFP fluorescence intensity in septal foci (mean ± STDEVP; *n* > 200 per strain, per time point; *p < 0.05, ****p < 0.0001, Mann–Whitney test) in the indicated strains at T2. **G** Immunoblot analysis of cell lysates containing CprV-GFP, collected at T2 and T3. The positions of CprV-GFP and GFP are indicated to the left and the position of molecular weight markers to the right. WT refers to the control strain lacking GFP. Asterisks indicate non-specific bands. **H** Bacterial two-hybrid (BTH) assays assessing interactions between CprV, SpoIIIE, SpoIIIM, and PbpG. Positive interactions are blue.

If CprV contributes to coordinating chromosome translocation and PG remodeling at the septal pore, it would be expected to localize in the septal membrane during the early stages of engulfment. To test this, we generated a CprV-GFP fluorescent fusion. CprV-GFP did not fully complement the Δ*cprV* Δ*spoIIIM* double mutant (Fig. S5C), suggesting that CprV-GFP is not fully functional. Thus, we examined CprV-GFP in a merodiploid background, which displayed wild-type levels of sporulation and showed identical localization as CprV-GFP expressed as the sole source of CprV (Fig. S5B, C). Consistent with the idea that CprV is a σ^E^-dependent protein, CprV-GFP localized primarily in the mother cell cytoplasm (Fig. 5A). However, in most cells we also observed a faint focus located at various positions in the engulfing membrane at T2 (Figs. 5A, B, and S5A). In some cells, we could clearly identify a CprV-GFP focus near the center of the septal membrane (9%), that we designated as a septal focus (Fig. 5B). At T3, we did not observe CprV-GFP foci (Fig. S5A). Interestingly, in a very small proportion of cells we observed a brighter CprV-GFP septal focus, and closer inspection of these cells revealed that they had septal defects (Fig. 5C). Thus, increased septal CprV localization appears to occur in response to septal defects. To further test this idea, we examined CprV-GFP localization in mutants with septal defects (Fig. 5A).

First, we examined CprV-GFP localization in Δ*pbpG* and Δ*spoIIIM* single mutants, which display low or moderate septal defects (and miscompartmentalization), respectively^30^. At T2, in both mutants, we observed an increased proportion of sporangia with a septal CprV-GFP focus, 10.5% and 16.8% for the Δ*pbpG* and Δ*spoIIIM*, respectively (Figs. 5A, E and S5A). Then, we examined the Δ*spoIIIM* Δ*pbpG* double mutant, which results in severe septal defects and miscompartmentalization. At T2, the double mutant exhibited a higher proportion of sporangia with a brighter CprV-GFP septal focus (58.4%) (Figs. 5A, E, and S5A). Quantification of CprV-GFP septal focus signal intensity in these mutants confirmed these observations (Fig. 5F). At T3, we observed fewer sporangia with a CprV-GFP focus in these mutants (Figs. S5A and S5D, E).

As the Δ*spoIIIM* Δ*pbpG* double mutant causes severe miscompartmentalization and chromosome translocation defects, CprV’s septal localization could be triggered by either of these defects. To distinguish between which defects lead to increased CprV-GFP septal localization, we examined a hypomorphic allele of SpoIIIE (SpoIIIE^D584A^), which results in a 2-fold decrease in chromosome translocation rate relative to WT^38,39^, and a low frequency of miscompartmentalization^30,31^ (17.9%; Fig. S4H, I). At T2, in SpoIIIE^D584A^ we observed a high proportion of sporangia containing a CprV-GFP septal focus (52.3%) (Figs. 5A, E, and S5F, G), with increased signal intensity relative to the WT (Fig. 5F). We also examined a SpoIIIE mutant that lacks the γ-domain (*spoIIIE*Δ*γ*), in which chromosome translocation rate is decreased 10-fold relative to WT^18,20^, and exhibits low levels of miscompartmentalization (12.5%; Fig. S4H, I). In *spoIIIE*Δ*γ* at T2, sporangia with a CprV–GFP septal focus were also frequent (57.6%; Fig. 5A, E) and had increased signal intensity compared to WT (Fig. 5F). Finally, we examined the *spoIIIE36* mutant, which results in a complete block to chromosome translocation^10,40^, but does impact compartmentalization^30,33^. Again, at T2, there was a higher frequency of sporangia with CprV-GFP septal localization (40.7%; Figs. 5A, E, and S5F, G) and increased focus signal intensity (Fig. 5F). As above, at T3, we observed fewer sporangia with a CprV-GFP focus in these mutants (Fig. S5A, D, E). Immunoblot analysis suggests that CprV-GFP focus frequency in the different mutants is not due to changes in protein levels, as CprV-GFP levels were similar between mutants (Fig. 5G). Thus, CprV-GFP localization as a septal focus is primarily triggered by chromosome translocation defects.

Next, we examined CprV-GFP localization in the absence of SpoIIIE. In this background, CprV-GFP failed to form a focus, bright or faint (Fig. 5D). To confirm these observations, we deleted *spoIIIE* in the Δ*spoIIIM* Δ*pbpG* double mutant which produces more frequent and brighter CprV-GFP foci (Fig. 5E, F). CprV-GFP failed to form a focus in the Δ*spoIIIE* Δ*spoIIIM* Δ*pbpG* triple mutant (Fig. 5D). Thus, CprV-GFP focal localization depends on SpoIIIE.

To investigate if SpoIIIE and CprV interact directly, we utilized bacterial two-hybrid assays in *Escherichia coli* (Fig. 5H). In this assay, *E. coli* strain BTH101 expresses SpoIIIE and CprV fusions to domains T25 and T18 of *Bordetella pertussis* adenylate cyclase^41^. Positive interactions between SpoIIIE and CprV rejoin the T25 and T18 domains, resulting in *lacZ* expression, β-galactosidase production and blue colonies on LB agar containing X-gal. Consistent with previous work and multimerisation of SpoIIIE^12,30^, we observed self-interaction between SpoIIIE-T25 and SpoIIIE-T18. We also observed self-interaction between CprV-T25 and CprV-T18, suggesting that CprV multimerizes. However, we failed to detect an interaction between SpoIIIE and CprV. Thus, although CprV’s focal localization in the septal membrane requires SpoIIIE, they may not interact in vivo. Additionally, we examined interactions between CprV and SpoIIIM, and CprV and PbpG, and could not detect interaction between these proteins.

Collectively, these data suggest that CprV localization at the engulfing septal membrane requires SpoIIIE and is primarily enhanced in response to chromosome translocation defects.

### CprV exhibits structural similarity to Alba proteins and likely multimerizes in vivo

**Fig. 6:**
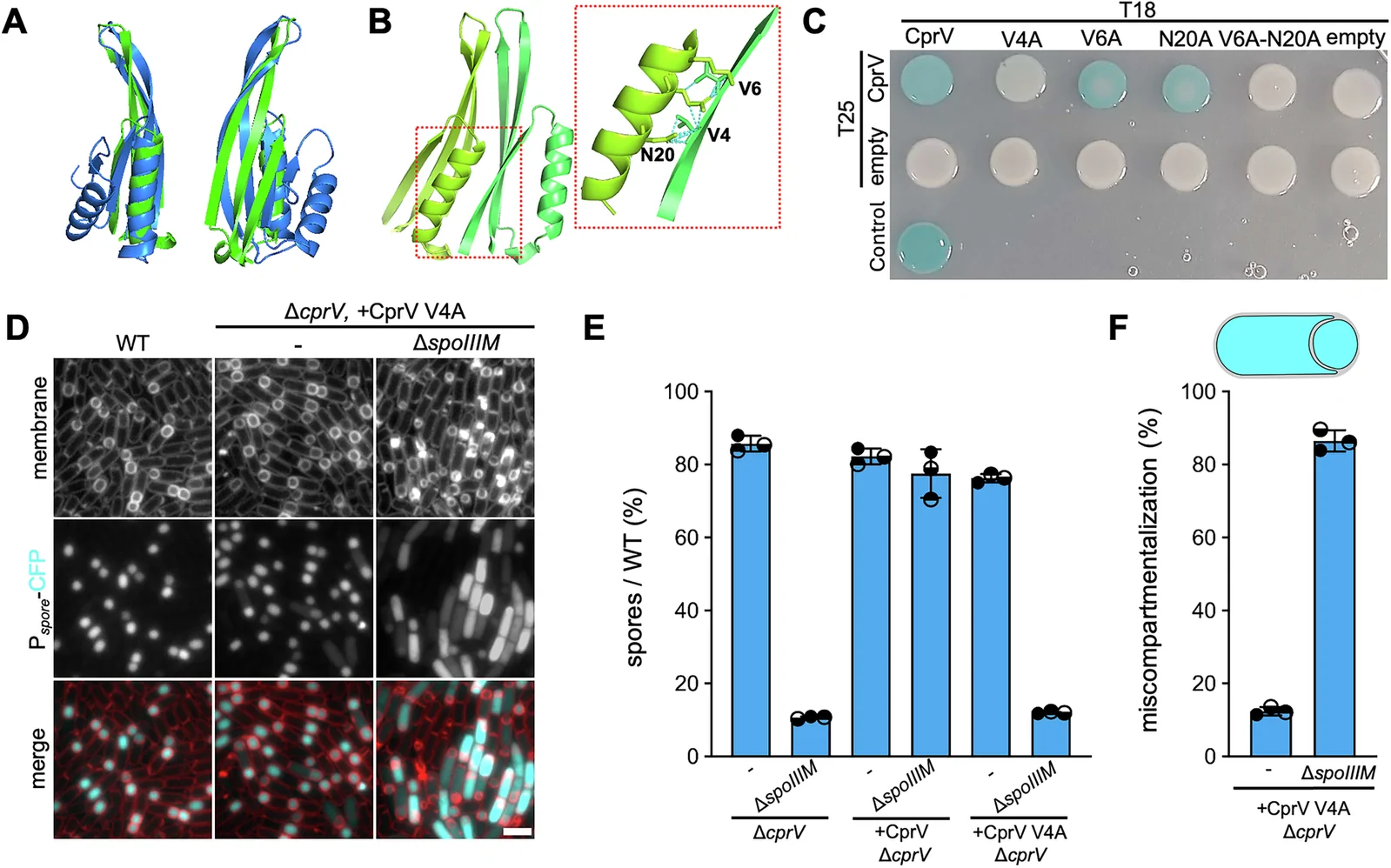
Characterization of CprV’s self-interaction. **A** Structural superposition of CprV (green) and the DNA/RNA-binding protein Alba1 (blue) from *Saccharolobus solfataricus* (UniProt: P60849; strain ATCC 35092), highlighting regions of structural similarity (TM-score: 0.64888; RMSD: 2.55). **B** Secondary structure and interaction interface of the CprV dimer, highlighting residues involved in intermolecular interactions within <3.5 Å, including V4, V6, and N20. **C** Bacterial two-hybrid (BTH) assays examining CprV dimerization using CprV mutant variants (V4A, V6A, N20A, and V6A/N20A) fused to T18, in combination with T25-fused wild-type CprV, to assess whether these mutations disrupt CprV–CprV interactions. T18-SpoIIIE and T25-SpoIIIE interaction is used as a positive control. **D** Representative microscopy images of *ΔcprV* and *ΔcprV ΔspoIIIM* strains complemented with CprV(V4A) at T3, showing that CprV(V4A) fails to complement the lack of *cprV* in either genetic background. Scale bar, 2 μm. **E** Average sporulation efficiency (% ± SD, *n* = 3) of Δ*cprV* and Δ*cprV* Δ*spoIIIM* strains complemented with CprV and CprV(V4A), showing that CprV(V4A) phenocopies Δ*cprV* and fails to restore sporulation, in either strain background. **F** Average frequency of miscompartmentalized cells at T3 (±SD of 3 biological replicates; *n* > 150 cells per strain, per time point, per replicate) in Δ*cprV*, Δ*cprV* Δ*spoIIIM* strains complemented with CprV(V4A), showing that CprV(V4A) fails to complement miscompartmentalization (for comparison refer to Fig. 1E for control strains).

To gain further insight into CprV function, we sought to identify structural similarity between CprV and other proteins. Using the Foldseek server^42,43^, we found that CprV exhibits structural similarity to Alba proteins found in archaea and eukaryotes (Fig. 6A). Alba proteins are typically dimeric and have been implicated in binding to nucleic acids, controlling a range of processes, from genome organization, transcriptional and translational regulation, RNA metabolism, and differentiation^44,45^. Interestingly, Alba proteins that bind nucleic acids are known to possess a basic isoelectric point, while Alba proteins that do not bind nucleic acids possess an acidic isoelectric point^46^. CprV possesses an acidic isoelectric point^34^, suggesting that it is unlikely to bind nucleic acids.

Our BTH data above suggest that CprV multimerizes. Consistent with this idea, Alphafold3 predicts various multimeric forms of CprV with high confidence (Fig. S6C). A CprV hexamer was particularly interesting due to its 6-fold symmetry supported by co-evolutionary analysis (Fig. S6A, B). Furthermore, computational simulations of the CprV hexamer suggest that it remains stable over a nanoscale time frame (Fig. S6D and Video S1). Analysis of the interface between two CprV molecules in the dimer (or hexamer) model revealed several residues that are likely to be important to promote CprV–CprV interactions (Fig. 6B). To test this, we used site-directed mutagenesis to introduce amino acid substitutions at specific residues located at the CprV–CprV interface (CprV^V4A^, CprV^V6A^, and CprV^N20A^) and examined the ability of these residues to maintain CprV–CprV interactions in the BTH assay. CprV^V4A^ showed a clear disruption of the CprV–CprV interaction, while CprV^V6A^ and CprV^N20A^ did not (Fig. 6C). Thus, we generated a CprV^V6A N20A^ double mutant, which led to the disruption of CprV–CprV interaction (Fig. 6C). Thus, CprV^V6A^ and CprV^N20A^ cooperate to maintain the CprV–CprV interaction.

To test the functional significance of the CprV–CprV interaction in vivo, we introduced the CprV^V4A^ mutant in the context of a functional C-terminal, hexahistidine-tagged version of CprV (CprV-His6) (Fig. 6E) at an ectopic locus in Δ*cprV* and Δ*cprV* Δ*spoIIIM* mutant strain backgrounds. We then examined the ability of the CprV^V4A^ mutant to complement sporulation and compartmentalization defects. CprV^V4A^ produced sporulation and compartmentalization defects that were identical to a Δ*cprV* mutant and failed to complement CprV in both backgrounds (Fig. 6D–F). For reasons unknown, we were unable to detect CprV-His6 with His6 antibodies by immunoblot and confirm the levels of CprV^V4A^. Thus, we tentatively conclude that multimerization is required for CprV’s role in compartmentalization.

Taken together, these data suggest that CprV may multimerize in vivo and that multimerization may be required for CprV’s role in compartmentalization.

### CprV is primarily found in the *Bacillales,* and its co-conservation with SpoIIIM and PbpG is species-specific

To examine the evolutionary distribution of CprV, we conducted a phylogenetic analysis across 497 representative *Firmicutes* genomes. The phylogenetic tree revealed that CprV is primarily found in the *Bacillales*, although it is occasionally found in a subset of *Clostridia* species (Fig. S7B). To gain further insight into the conservation and co-conservation of SpoIIIE, CprV, SpoIIIM and PbpG, we also examined their distribution across the 497 *Firmicutes* genomes (Fig. S7A, B). As expected, SpoIIIE is widely conserved across *Firmicutes,* likely due to its essential role in chromosome translocation. Although PbpG has a more limited conservation than SpoIIIE, it was found in various orders but with less frequent conservation in *Clostridia*. On the other hand, SpoIIIM was exclusively found in the *Bacillales*. Thus, the co-occurrence of all four factors is restricted to a subset of *Bacillales* species. Together, these observations suggest that the mechanisms controlling coordination between chromosome translocation and PG remodeling, and maintenance of compartmentalization, differ in complexity across spore-forming *Firmicutes*.

## Discussion

Molecular mechanisms that maintain cellular compartmentalization are essential for successful differentiation and development across life. Our data demonstrate that in addition to SpoIIIM and PbpG, CprV supports efficient coordination between chromosome translocation and PG remodeling during engulfment, which in turn is important for ensuring septal pore stability and developmental compartmentalization during sporulation. Our data indicate that the absence of CprV alone results in relatively mild defects; however, when SpoIIIM or PbpG are absent, CprV becomes critical, with Δ*cprV* Δ*spoIIIM* and Δ*cprV* Δ*pbpG* double mutants displaying severe, synthetic defects in sporulation, forespore morphology, chromosome translocation and compartmentalization. Furthermore, we show that CprV localizes at the septal pore in a SpoIIIE-dependent manner and that its localization there increases in frequency in response to chromosome translocation defects. The significance of CprV’s localization and its function in maintaining coordination between chromosome translocation and PG remodeling are discussed.

How does CprV localize to the septal pore? Our data suggest that CprV localization at the septal pore is not due to miscompartmentalization per se, but rather due to defects in chromosome translocation. Indeed, we showed that chromosome translocation mutants (*spoIIIE36*, SpoIIIE^Δγ^, and SpoIIIE^D584A^) with limited effect on compartmentalization result in increased septal focal localization of CprV (Fig. 5E). Interestingly, even though CprV localization as a focus is SpoIIIE-dependent (Fig. 5D), unlike SpoIIIM and PbpG, it does not appear to interact with SpoIIIE directly, at least by bacterial two-hybrid (Fig. 5H). Thus, CprV’s focal localization may require an additional factor that interacts with SpoIIIE at the septal pore. Alternatively, CprV may sense a structural feature of the septal pore that requires SpoIIIE and is altered in mutants that compromise chromosome translocation. Although the ultrastructure of the septal pore remains unclear, as is the exact arrangement of SpoIIIE at the septal pore, using computational modeling and co-evolutionary analysis, we modeled a toroidal membrane pore including two SpoIIIE hexameric motor domains (Fig. S7C–G). In this model, two SpoIIIE hexameric motor domains are joined by their transmembrane domains to form a transmembrane domain 12-mer that circumferentially surrounds a toroidal membrane pore delineated by PG resulting from incomplete cytokinesis over the partially segregated chromosome (Fig. S7H, I). While this computational model does not fully consider the number of SpoIIIE molecules present in the septal focus as reported by photoactivated localization microscopy (34–47 molecules)^35,36^, it is likely that mutants impacting septal pore stability or chromosome translocation affect the structural architecture of the septal pore. Thus, CprV may recognize a physical change in the membrane surrounding the septal pore (e.g., membrane tension), or the extreme positive membrane curvature of the septal pore, and reinforce the septal pore in response to chromosome translocation defects. Proteins that localize to the membrane in response to changes in membrane tension or membrane curvature are well known in prokaryotic and eukaryotic biology^47–49^.

What is the exact role of CprV in supporting the coordination between chromosome translocation and PG remodeling at the septal pore and compartmentalization? Although blocking engulfment PG hydrolysis suppresses the Δ*cprV* defects, CprV, unlikely counterbalances DMP complex activity at the septal pore by facilitating SpoIIIE-PG interactions, like SpoIIIM^30^, or by building new PG around the pore, like PbpG^30^. Instead, CprV’s role in this coordination mechanism appears linked to chromosome translocation, and several observations are consistent with this idea. First, while there was a marginal increase in miscompartmentalization in the Δ*cprV* Δ*spoIIIM* Δ*pbpG* triple mutant compared to Δ*spoIIIM* Δ*pbpG* double mutant (Fig. 4B), we observed a more severe chromosome translocation defect in the Δ*cprV* Δ*spoIIIM* Δ*pbpG* triple mutant than the Δ*spoIIIM* Δ*pbpG* double mutant (Fig. 4E, F); thus, CprV function appears to be more strongly linked to promoting chromosome translocation than compartmentalization. Second, CprV localization at the septal pore appears to occur in response to chromosome translocation defects, rather than compartmentalization defects per se: we observed increased CprV-GFP septal focus localization in various SpoIIIE mutants that are only mildly impacted in compartmentalization or not impacted at all (Fig. 5A, E). Thus, although CprV does not appear to interact with SpoIIIE, its function in maintaining coordination between chromosome translocation and PG remodeling and compartmentalization appears tied to SpoIIIE’s activity in chromosome translocation.

**Fig. 7:**
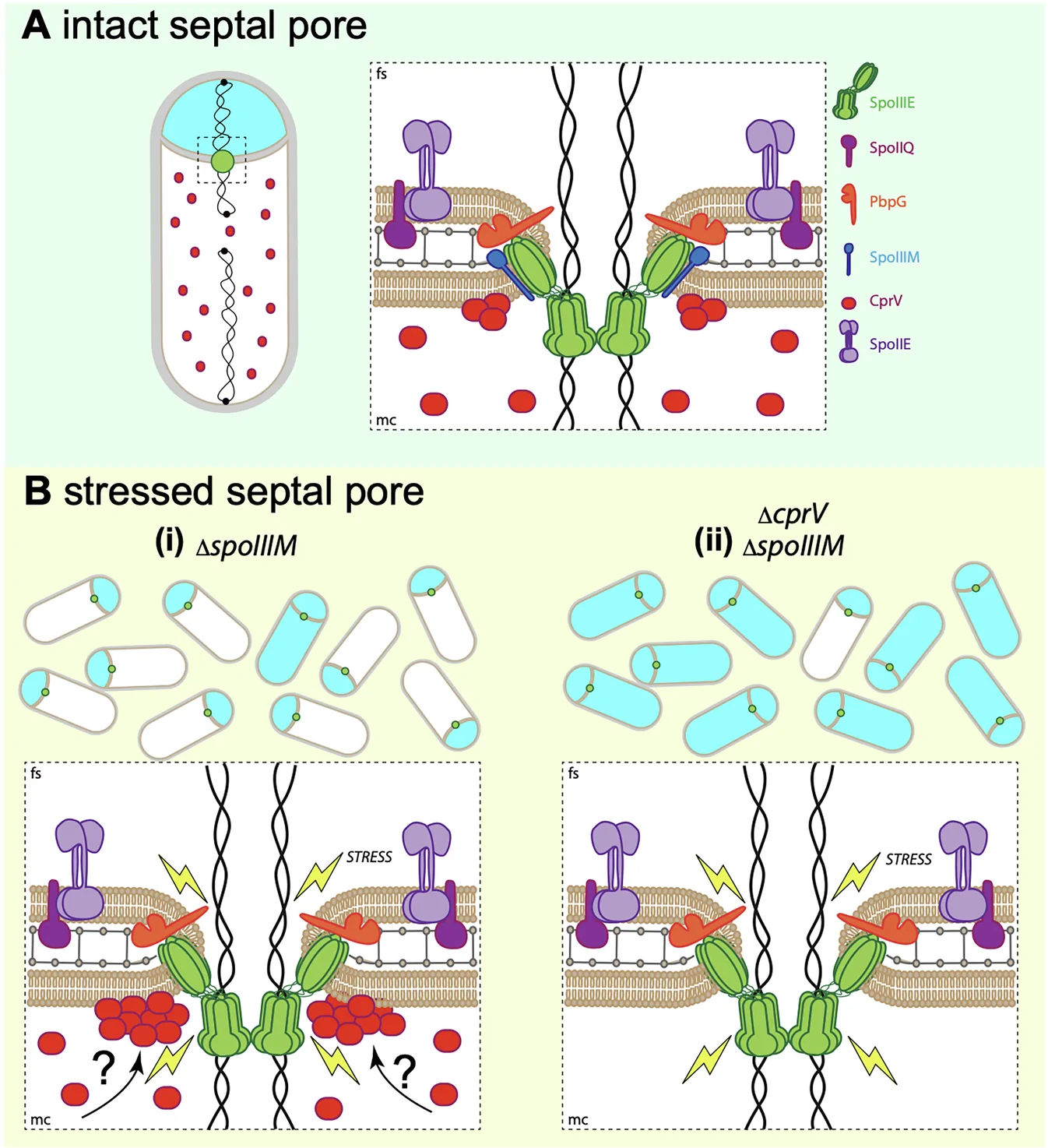
Hypothetical model illustrating CprV’s safeguarding function at the septal pore. **A** In developing cells that efficiently coordinate chromosome translocation with PG remodeling and maintain compartmentalization (cyan) at the stable septal pore, CprV is residually localized in the engulfing membrane as a focus. **B** In response to defects in septal pore stability (yellow lightning-bolt indicating stress) (e.g., Δ*spoIIIM* mutant), CprV multimerization at the septal pore increases, where it contributes to septal pore stability, efficient chromosome translocation and compartmentalization (i). When other septal pore stabilization factors are missing (e.g., Δ*spoIIIM* mutant), the absence of CprV exarcerbates septal pore instability (yellow lightning-bolt indicating stress), leading to increased miscompartmentalization (cyan) and chromosome translocation defects (for simplicity not shown) (ii).

While the exact mechanism of how CprV supports septal pore stability remains unclear, the above observations point to the possibility that SpoIIIE activity influences septal pore stability and CprV reinforces septal pore stability in response to changes in SpoIIIE activity. How CprV responds to SpoIIIE activity is likely indirect, and could involve an intermediate factor, as hypothesized above, or biophysical effects, such as changes in membrane tension at the septal pore that result from SpoIIIE translocating DNA at slower speeds. Interestingly, our computational modeling and co-evolutionary analysis suggest that CprV multimerises (Fig. S6) and this multimerisation may be required for CprV function, including compartmentalization (Fig. 6D–F). Thus, CprV multimerisation could be required to reinforce the membrane surrounding the septal pore to ensure SpoIIIE retains its ability to coordinate chromosome translocation and PG remodeling, and therefore maintain compartmentalization, particularly when other septal pore stability factors are missing. Based on these observations, we propose a model whereby in WT cells, CprV acts as a safeguarding factor (Fig. 7). CprV is residually localized in the engulfing membrane, and when cells experience chromosome translocation defects, CprV becomes enriched at the septal pore to stabilize the architecture of the septal pore, which in turn supports SpoIIIE’s role in coordinating chromosome translocation and PG remodeling (Fig. 7A). In conditions where septal pore stability is already compromised, such as the absence of SpoIIIM or PbpG, CprV provides additional stability to the septal pore to prevent worsening of chromosome translocation and compartmentalization defects (Fig. 7B). The additional stability CprV provides to the septal pore was also apparent when septal PG hydrolysis was blocked in cells lacking PbpG, SpoIIIM and PbpG, or SpoIIIM, PbpG and SpoIIQ. In these mutants CprV appears to be essential for maintaining compartmentalization, as blocking septal PG hydrolysis was insufficient to restore compartmentalization to all cells (Fig. S2A, B). While these data suggest that CprV functions to safeguard septal pore stability, it is formerly possible that CprV functions to prevent ectopic σ^F^ activation in specific mutant backgrounds. However, since σ^F^ activation is controlled by SpoIIE in the forespore and CprV is transcribed in the mother cell, we do not favor this model.

An intriguing observation we made previously^30^, and observed again in this study, relates to SpoIIIE’s localization as the focus in the engulfing membrane. While the SpoIIIE-GFP focus in WT sporangia dissipates as they approach engulfment completion, the focus persists in various mutants defective in coordinating chromosome translocation and PG remodeling (Figs. 2B and 4H). Since SpoIIIE focus formation depends on DNA passing through the septum and is thought to represent SpoIIIE complexes bound to DNA^37^, SpoIIIE likely remains bound to DNA in mutants, whereas in WT sporangia it dissociates. Since the various mutants tested are defective in the coordination between chromosome translocation and PG remodeling, it is possible that coordination between these processes is what allows SpoIIIE to detach from DNA, once chromosome translocation is completed and septal pore closure occurs. How chromosome translocation and PG remodeling are biochemically connected remains unclear. Future work examining this question could reveal how SpoIIIE detaches from DNA in response to septal pore closure.

Looking more broadly at how chromosome segregation and PG remodeling are coordinated during sporulation in other species, our phylogenetic analyses suggest that only a subset of spore-forming species employ all three proteins, CprV, SpoIIIM and PbpG, to work in concert with SpoIIIE (Fig. S7B). Thus, some species have evolved alternative mechanisms to sustain septal pore stability and compartmentalization during chromosome translocation and engulfment. Previously, it was shown that reducing the activity of the engulfment PG hydrolases restores compartmentalization in the absence of SpoIIIM and PbpG^30^. Therefore, it is possible that some species evolved slowly acting septal PG hydrolases, which would compromise engulfment speed but allow maintenance of compartmentalization at the septal pore during engulfment without the need for additional factors. It would be interesting to examine the relationship between chromosome translocation and PG remodeling in *Clostridia*, where SpoIIIM, PbpG are lacking, and CprV is rare.

Overall, this study provides deeper insight into the mechanisms and associated factors that maintain compartmentalization during bacterial sporulation. Future work directed at the identification of the molecular or biophysical signals that recruit CprV to the septal pore, and the molecular architecture of the septal pore and its components, could reveal the finer mechanisms of how these factors cooperatively function to maintain efficient coordination between chromosome translocation and PG remodeling, and compartmentalization.

## Methods

### General methods

Strains, plasmids, and oligonucleotide primers (sourced from IDT) used in this study are listed in Table S1. All *Bacillus subtilis* strains used in this study were derived from the auxotrophic strain 168. Sporulation was induced either by resuspension at 37 °C following the Sterlini–Mandelstam protocol^50^ or by nutrient depletion in a supplemented Difco sporulation medium (DSM)^51^. The DSM medium contained 8 g/L bacto nutrient broth (Difco), 0.1% (wt/vol) KCl, 1 mM MgSO_4_, 0.5 mM NaOH, 1 mM Ca(NO_3_)_2_, 0.01 mM MnCl_2_, and 0.001 mM FeSO_4_. Sporulation efficiency was assessed using a heat-resistance assay. Cultures grown in DSM for 30 h at 37 °C were subjected to heat treatment at 80 °C for 20 min, and the number of heat-resistant colony-forming units (CFUs) was compared to that of the wild-type strain.

### Transposon insertion sequencing

Transposon insertion sequencing (Tn-seq) adapted for sporulation^52^ was performed on the wild-type strain bDR2413 and the Δ*spoIIIM* mutant (bAT17). Approximately 750,000 transformant colonies were harvested, aliquoted, and frozen. An aliquot was thawed on ice, resuspended in DSM, and diluted into 50 mL of DSM to an OD_600_ of 0.05. Samples were collected at 24 h (T24). T24 samples were incubated at 80 °C for 20 min to eliminate sporulation-defective cells and were then plated on LB agar to allow germination and outgrowth of heat-resistant, germination-proficient spores. Approximately 750,000 colonies were subsequently harvested, and genomic DNA was extracted and digested with *Mme*I, which cuts at the *Mme*I restriction site introduced within the inverted repeat sequences of the magellan6 transposon. Barcode adapters were ligated, and transposon–chromosome junctions were amplified by PCR using 18 cycles. PCR products were gel-purified and sequenced on an Illumina MiSeq platform using MiSeq reagents. Sequencing reads were mapped to the *Bacillus subtilis* 168 genome (NCBI NC_000964.3) and tallied at each TA insertion site. Genes with statistically underrepresented insertions were identified using the Mann–Whitney *U* test. Transposon insertion profiles were visually inspected using the Sanger Artemis Genome Browser and Annotation Tool.

### Live-cell fluorescence imaging

Live-cell fluorescence imaging was performed by placing cells on a 2% (w/v) agarose pad prepared with resuspension medium and mounting them on a Gene Frame (Thermofisher). For sporulating cultures obtained via the resuspension method, 250 µL of culture was collected at the designated time point, pelleted by centrifugation, and resuspended in 10 µL of resuspension medium containing 0.05 mM TMA-DPH [1-(4-trimethylammoniumphenyl)-6-phenyl-1,3,5-hexatriene p-toluenesulfonate]. A 2 µL aliquot of the stained cell suspension was then spread onto the agarose pad, and a coverslip was placed on top of the adhesive Gene Frame.

Cells were imaged using a Zeiss Axio Observer 7 microscope equipped with a Plan-Apochromat 100×/1.4 oil Ph3 objective lens and a Colibri 7 type R[G/Y]CBV-UV fluorescent light source. Images were captured with a Zeiss Axiocam 712 mono camera. The TMA-DPH membrane dye and GFP were excited using a Zeiss Axio 92HE filter with 100 and 300 ms exposure time, respectively. CFP fluorescence was acquired using a Zeiss Axio 108HE filter (150 ms exposure).

SIM imaging was performed using a Zeiss Elyra 7 wide-field high-resolution system equipped with a PCO Edge 4.2 sCMOS camera. Coherent laser sources at 488 nm (100 mW) and 561 nm (100 mW) were used to excite CprV-GFP and the FM4-64 membrane dye, respectively. The exposure time for SIM acquisition was set to 80 ms.

### Image processing and quantitative image analysis

Image post-processing was performed using Fiji software (version 2.14.0/1.54f)^53^ to adjust brightness and contrast. Quantitative analyses were carried out using the manual counting tool in Fiji, and the resulting data were exported to Microsoft Excel (version 16.83) and GraphPad Prism (version 10.2) for compilation and graph generation.

For quantification of the number of sporangia containing a CprV-GFP focus localized in mother-cell proximal region of the engulfing membrane, the frequency of cells containing this localization was determined as a percentage of the total number of cells exhibiting detectable GFP fluorescence. Additionally, the point tool in Fiji software was used to quantify the intensity of CprV-GFP fluorescence within these foci. For statistical analysis, the mean fluorescence intensity values for each focus were extracted.

SpoIIIE-GFP fluorescence intensity was quantified using the MicrobeJ plugin for the Fiji software^54^. Images were first processed by subtracting background fluorescence (*Process* *>* *Subtract Background*) to minimize false-positive signal detection. The “bacteria” tab in MicrobeJ was then used to identify sporangial outlines from phase-contrast images, with the “smoothed” option enabled. The parameters “exclude on edges,” “shape descriptors,” and “segmentation” were selected to refine object detection. To identify SpoIIIE-GFP foci and measure mean fluorescence intensity, the “maxima” tab in MicrobeJ was configured to the “Point” setting.

For quantification of the miscompartmentalization defect, cells showing CFP fluorescence originating from the forespore but appearing in the mother cell, while also containing a visible spore compartment (including deformed spores), were classified as miscompartmentalized.

For quantification of septal retraction, cells displaying CFP fluorescence from the forespore in the mother cell, accompanied by either a discontinuous polar septum or an absence of a visible septum, were classified as having a retracted septum. In both cases, the frequencies of miscompartmentalization and septal retraction were calculated relative to the total number of cells exhibiting CFP fluorescence (i.e., cells in which σ^F^ was active).

### Immunoblot analysis

Immunoblot was conducted following the methodology in Luhur et al.^55^. Briefly, whole-cell lysates from sporulating cells were heated for 5 min at 90 °C. Equivalent loading was based on the OD_600_ at T2 and T3. Samples were separated on a 10% polyacrylamide gel and transferred to a PVDF membrane. Membranes were blocked in 5% non-fat milk powder in PBS, with 0.5% Tween-20 for 1 h. Blocked membranes were probed with anti-GFP (1:10,000) (Abcam) primary antibody diluted into PBS with 0.05% Tween-20 containing 5% non-fat milk powder (w/v) at 4 °C overnight. Primary antibodies were detected with horseradish-peroxidase anti-rabbit antibodies (BioRad) and detected with ECL Prime Western Blotting Detection reagent (Amersham) as described by the manufacturer.

### Computational simulations

CprV hexamers were modeled with AlphaFold3^56^, using the full amino acid sequence of the *Bacillus subtilis* str. 168 protein (UniProt P94496**)**. The full atom model was then simulated using GROMACs^57^ charmm36 force fields for 100 ns at 310 K, in 200 mM NaCl at pH 7 using CHARMM-GUI soluble protein (https://www.charmm-gui.org/) inputs. Simulations were visualized in PyMOL. Illustrative models of Toroidal membranes were modeled using MemTorMD coarse grain simulations^58^.

### Co-evolutionary contacts analysis

Co-evolutionary contacts within CprV and SpoIIIE were calculated using the GREMLIN algorithm^59^ using CoEVRank^60^ with a MMSEQS2 cutoff of >50% and no identity cutoff, uncovering 600 amino sequences for alignment. To generate contact maps displaying intermolecular co-evolving contacts, we used the AlphaFold3 structures of the CprV monomer, trimer and hexamer or SpoIIIE transmembrane domain as a monomer, hexamer and dodecamer as inputs. The contact map was set such that interacting residues at the protomer interface are within 12 Å from the amino acid carbon backbone.

### Phylogenetic analyses

We assembled a local database of *Firmicutes* containing 497 genomes and inferred the corresponding reference phylogenetic tree using the approach in Chan et al.^29^. To infer sporulating taxa in the *Firmicutes*, we searched for homologs of Spo0A against our Firmicutes database using the pfam domain PF08769 (Spo0A_C) and the HMMSEARCH tool from the HMMer package^61^ with the option cut_ga.

To search for homologs of PbpG, SpoIIIE, CprV and SpoIIIM proteins in the *Firmicutes* database, we first scanned *Bacillus subtilis* corresponding protein sequences BSU_37510, BSU_16800, BSU_30080 and BSU_25060 for Pfam domains^62^, and identified the presence of the domains PF00912 and PF00905 in PbpG (BSU_37510), PF01580 in SpoIIIE (BSU_16800) and PF10957 in CprV (BSU_30080). As no domain was detected in SpoIIIM (BSU_25060), a first round of homology search was run using blastp^63^ against the Firmicutes database. All hits with an *e*value < 1e-03 were next extracted and aligned using MAFFT (with the linsi option)^64^, and an HMM profile was built using HMMBUILD. Iterative homology searches of the four proteins were run against the Firmicutes database using HMMSEARCH. For each protein family, all hits were retrieved and curated using alignments, domain organization and phylogeny to detect and remove false positive hits. Single gene phylogenies were run using IQ-Tree2^65^. All phylogenies were annotated using IToL^66^.

### Statistics and reproducibility

All statistical analyses were performed using non‑parametric methods unless otherwise stated. For comparisons between two groups, the Mann–Whitney test was used. Data are presented as mean ± SD for experiments with biological replicates, and exact *p* values are reported in the figure legends. No statistical methods were used to predetermine sample size, and no data were excluded. Experiments were not randomized, and investigators were not blinded to sample allocation. Experiments were performed with three biological replicates, unless otherwise stated, each starting from an independent culture that was grown and analysed separately. The number of cells analysed for each replicate and condition is reported in the corresponding figure legends.

### Inclusion and ethics

The authors are committed to developing research in an ethical, rigorous, and inclusive manner. This study did not involve human participants, human tissues, personal data, or vertebrate animals. All experiments were performed in accordance with institutional guidelines and relevant regulations for the safe handling of biological materials. The authors have sought to foster an inclusive and equitable research environment throughout the development of this work. Contributions were recognized based on scientific input, without discrimination based on gender, ethnicity, nationality, disability, career stage, or other protected characteristics. The study was designed, developed, and reported in accordance with principles of research integrity, transparency, and reproducibility.

### Reporting summary

Further information on research design is available in the Nature Portfolio Reporting Summary linked to this article.

## Supplementary information

Transparent Peer Review File

Supplementary Information

Description of Additional Supplementary Files

Supplementary Data

Supplementary Video 1

Reporting Summary

## Data Availability

Supplementary Data 1 includes numerical source data presented in graphs, and Tn-seq data used in the identification of top hits can be found here at https://doi.org/10.6084/m9.figshare.31890352. Supplementary Video 1 (Video S1) can be found here at https://doi.org/10.6084/m9.figshare.31971588. Uncropped and unedited blot images shown in Fig. 5G are shown in Supplementary Fig. 8. All other source data, including plasmids, are available from the corresponding author on reasonable request.
