# Supplementary material for: A safeguarding factor monitors septal pore stability to secure compartmentalization during sporulation in Bacillus subtilis
Source: Commun Biol. 2026 Jul 15;9:1271. doi: 10.1038/s42003-026-10705-y (PMC13627676; doi:10.1038/s42003-026-10705-y)
Supplement: Supplementary file 1 [file 42003_2026_10705_MOESM1_ESM.pdf]

nature portfolio

Peer Review File

A Safeguarding Factor Monitors Septal Pore Stability to Secure Compartmentalization during Sporulation in Bacillus subtilis

Corresponding Author: Dr Christopher Rodrigues

This file contains all reviewer reports in order by version, followed by all author rebuttals in order by version.

Version 0:

Reviewer comments:

Reviewer #1

(Remarks to the Author)
A particular challenge facing sporulating bacterial cells of the Bacillales concerns the maintenance of distinct cytoplasmic compartmentalisation between forespore and mother cell while attempting to thread DNA from the latter compartment to the former. This occurs while septal PG is being remodelled during the process of engulfment, hence compartmentalisation has to be maintained essentially while there is a channel connecting the two cellular compartments. Most if not all of the major protein players in this scheme have been identified previously, although heterogeneity within sporulating cultures indicate that the story is incomplete. This is where the current manuscript picks up. Starting with a transposon led screen conducted in an appropriate mutant background the authors identify CprV as a new (arguably secondary) component that is involved in the maintenance of compartmentalisation during sporulation. More specifically, as identified via a comprehensive series of elegantly designed experiments, the authors identify CprV as having a safeguarding role during DNA translocation, assisting with the maintenance of septal spore stability in tandem with SpoIIIM and PbpG (indeed, the significance of CprV was only teased out in strains with double and triple mutants in these genes). Intriguingly, the protein appears to multimerise in a stress dependent manner (i.e., stress associated with DNA translocation defects or in the absence of septal pore stability factors) at or near SpoIIIE at the septal pore, but the two proteins do not physically interact (hence, there may be additional players yet to be identified). The authors suggest that the stress sensing response might be mediated by changes in forespore membrane tension or curvature and that CprV multimerization might serve in a structural role to stabilise the pore, which sounds plausible. The protein bears structural similarity to DNA-binding Alba proteins but the authors suggest that CprV neither binds DNA nor is dimeric, presenting a predicted hexameric arrangement that is supported by MD simulations and mutagenesis data.

Overall the work has been conducted to an extremely high standard. The paper is equally well written and presented with a compelling narrative that should appeal to expert and non-specialist readers alike. The results are novel and place an additional important piece of the jigsaw to the sporulation puzzle.

I have only a few minor typos that should be addressed:

Line 50 – founds/found, delete the comma
Line 232 – add mutant
Line 250 – of SpoIIIE
Line 450 – remove italics from ‘in’, add to lacZ.
Figure 3 legend – ‘in each background’ is duplicated.
Figure 4 heading – dynamics
Figure 7 – SpoIIIM protein is still evident in Figure B.
Bibliography – Ref 36 has a typo.

Reviewer #2

(Remarks to the Author)
In this article, Dehghani et al. report the characterization of a novel sporulation gene that they rename cprV. About 30 years ago, this gene was originally annotated as yteV and shown to be expressed under the control of the sporulation sigma factor

SigE. Yet, its role in sporulation had remained mysterious. Here, the authors show that redundancy played a major role in masking its function. It is only when a second sporulation gene, spoIIIM (yqfZ), is missing that a clear phenotype emerges in sporulating cells. Dehghani et al. performed Tn-seq experiments to identify cprV in the sensitized spoIIIM deletion mutant background. They performed several assays to show that compartmentalization, septal integrity, chromosome translocation (and retention in the forespore) are impaired in a spoIIIM cprV double mutant and even more drastically in a spoIIIM cprV pbpG triple mutant. In addition, they show that the DNA translocase SpoIIIE fused to GFP persists longer than in the wild type strain in the absence of cprV. Recruitment of CprV to the septal pores appears to be dependent on SpoIIIE, although probably not by direct interaction. They also show that based on similarity to the Alba family of DNA/RNA binding proteins, CprV likely forms a hexamer and identify amino acids necessary for multimerization. There are still a few minor gaps in the model regarding the recruitment of CprV to the septal pore, but on the whole the model presented in Fig.7 is supported by solid experimental evidence.
I only have a few minor suggestions listed below.
Abstract: Add the original gene names (yteV, cse60) and the definition of the acronym in the abstract.
I agree that it is probably a good idea to rename the gene, because yteV is too cryptic; however, I don't completely follow the logic of calling this gene cprV (why V?). Also, previously, when the same group identified YqfZ as another compartmentalization protein, they renamed it SpoIIIM. Wouldn't it make more sense to rename YteV as SpoIIIN? Or SpoIIIM as CprZ?
l.122-3 "and where the highly conserved SpoIIE-SpoIIQ interaction is abolished,". This concept requires some introduction as SpoIIE was not mentioned in the introduction and can easily be confused with SpoIIIE by readers who are not experts in the sporulation field.
l.181-3 "smaller forespores or forespores with irregular membranes (Fig. 1D)." The image shown is a bit small and too crowded to unambiguously visualize this. Maybe show a few zoomed in examples in the supplement.
l. 218-219 "although to a lesser extent that SpoIIIM". "than" not "that"
Fig. 4A and B, I found the legend on the y axis to be a bit confusing. It is supposed to be a comparison of the double mutant to the triple mutant, but I originally read it as a comparison of a spoIIIM deletion to cprV pbpG double mutant.

Reviewer #3

(Remarks to the Author)
In Bacillus subtilis, DNA translocation during sporulation occurs through a specialized septal pore that forms during polar septation over the partially segregated forespore chromosome. This process is tightly coordinated with septal peptidoglycan (PG) remodeling to preserve compartmentalization between the mother cell and forespore. Central to this coordination is a complex comprising the DNA translocase SpoIIIE, the forespore-produced PG synthase PbpG, and the mother-cell protein SpoIIIM, which together maintain septal pore stability by balancing PG synthesis and hydrolysis. This balance ensures that the pore remains sufficiently large to allow DNA passage while remaining constrained enough to prevent cytoplasmic leakage.
Loss of SpoIIIM and PbpG significantly affects compartmentalization but not spore formation. In contrast, in the absence of SpoIIIE, all cells become miscompartmentalized and no spores are formed. These observations suggest that additional SpoIIIE-dependent factors contribute to septal pore stability and compartmentalization.
This study identifies CprV as a new protein involved in maintaining gene-expression compartmentalization during sporulation in response to septal pore stability defects. Although the cprV single mutant displays only mild defects in compartmentalization and chromosome translocation, combining cprV deletion with mutations in spoIIIM and/or pbpG exacerbates these phenotypes. These double and triple mutants show reduced formation of heat-resistant spores, altered SpoIIIE localization dynamics, defects in chromosome translocation into the forespore, and increased miscompartmentalization of gene expression. CprV is a small 60 amino acid protein with predicted structural similarity to Alba proteins. The authors show that CprV can self-interact, and in silico predictions suggest that it may form a hexamer. Substitutions in residues located at the predicted monomers interface disrupt CprV self-interaction and impair its function in vivo.
Overall, the manuscript is clearly and convincingly presented. The experiments are rigorous and, in general, well interpreted, and the study represents an important contribution to the field. I have only a few suggestions that may help improve the manuscript.

Main comment: It remains somewhat difficult to distinguish miscompartmentalization caused by cytoplasmic leakage from ectopic activation of compartment-specific sigma factors. The cprV single mutant also shows some degree of miscompartmentalization; does this imply that septal pore stability is compromised even in this background? If so, could this be directly assessed, for example by electron microscopy or another approach capable of visualizing septal pore architecture? This point seems particularly relevant because, unlike the suppression observed upon deletion of spoIID and spoIIP in other septal pore stability mutants, loss of PG hydrolysis in the cprV mutant appears to suppress chromosome translocation defects but not the miscompartmentalization of gene expression. An alternative possibility is that CprV may have a more direct role in regulating compartment-specific gene expression. Given its predicted structural similarity to Alba proteins, despite its acidic nature, it would be useful to discuss whether CprV could influence gene expression independently of, or in addition to, effects on septal pore stability.

Line 78: consider revising "σF, σE, σG and σK" to superscript throughout the manuscript

Lines 489–494: It would be important to confirm that the CprVV4A allele accumulates to levels comparable to wild-type CprV in Bacillus subtilis cells. Without this control, the conclusion that loss of function is specifically due to impaired multimerization should be made with caution, as the V4A substitution could also affect protein stability, expression, or

accumulation.

Figure 1 legend: “(refs)” is still present in the figure legend and should be replaced with the appropriate references.

Version 1:

Reviewer comments:

Reviewer #2

(Remarks to the Author)
I believe that the authors have addressed all the comments satisfactorily. There is just one response that I disagree with. I still think that it would be helpful to have the original name of the gene (yteV) mentioned in the abstract. I know from personal experience that literature searches in various databases are greatly facilitated when synonyms are clearly spelled out. I also don't think that word count is a valid argument to deny this request as it only adds one word to the abstract.

Reviewer #3

(Remarks to the Author)
The original manuscript was excellent. Only minor revisions were requested, and the authors have in same way addressed them.

Open Access This Peer Review File is licensed under a Creative Commons Attribution 4.0 International License, which permits use, sharing, adaptation, distribution and reproduction in any medium or format, as long as you give appropriate credit to the original author(s) and the source, provide a link to the Creative Commons license, and indicate if changes were made.
In cases where reviewers are anonymous, credit should be given to 'Anonymous Referee' and the source.
The images or other third party material in this Peer Review File are included in the article’s Creative Commons license, unless indicated otherwise in a credit line to the material. If material is not included in the article’s Creative Commons license and your intended use is not permitted by statutory regulation or exceeds the permitted use, you will need to obtain permission directly from the copyright holder.
To view a copy of this license, visit https://creativecommons.org/licenses/by/4.0/

Dehghani et al – Response to Reviewer Comments (in blue)

Reviewer #1 (Remarks to the Author):

A particular challenge facing sporulating bacterial cells of the Bacillales concerns the maintenance of distinct cytoplasmic compartmentalisation between forespore and mother cell while attempting to thread DNA from the latter compartment to the former. This occurs while septal PG is being remodelled during the process of engulfment, hence compartmentalisation has to be maintained essentially while there is a channel connecting the two cellular compartments. Most if not all of the major protein players in this scheme have been identified previously, although heterogeneity within sporulating cultures indicate that the story is incomplete. This is where the current manuscript picks up. Starting with a transposon led screen conducted in an appropriate mutant background the authors identify CprV as a new (arguably secondary) component that is involved in the maintenance of compartmentalisation during sporulation. More specifically, as identified via a comprehensive series of elegantly designed experiments, the authors identify CprV as having a safeguarding role during DNA translocation, assisting with the maintenance of septal spore stability in tandem with SpoIIIM and PbpG (indeed, the significance of CprV was only teased out in strains with double and triple mutants in these genes). Intriguingly, the protein appears to multimerise in a stress dependent manner (i.e., stress associated with DNA translocation defects or in the absence of septal pore stability factors) at or near SpoIIIE at the septal pore, but the two proteins do not physically interact (hence, there may be additional players yet to be identified). The authors suggest that the stress sensing response might be mediated by changes in forespore membrane tension or curvature and that CprV multimerization might serve in a structural role to stabilise the pore, which sounds plausible. The protein bears structural similarity to DNA-binding Alba proteins but the authors suggest that CprV neither binds DNA nor is dimeric, presenting a predicted hexameric arrangement that is supported by MD simulations and mutagenesis data.

Overall the work has been conducted to an extremely high standard. The paper is equally well written and presented with a compelling narrative that should appeal to expert and non-specialist readers alike. The results are novel and place an additional important piece of the jigsaw to the sporulation puzzle.

R: We thank the reviewer for their positive assessment and for recognizing the value of our work. We appreciate their time during peer review.

I have only a few minor typos that should be addressed:

Line 50 – founds/found, delete the comma
R: This has been amended

Line 232 – add mutant
R: This has been added

Line 250 – of SpoIIIE
R: This has been amended

Line 450 – remove italics from ‘in’, add to lacZ.
R: This has been amended

Figure 3 legend – ‘in each background’ is duplicated.
R: This has been amended

Figure 4 heading – dynamics
R: This has been amended

Figure 7 – SpoIIIM protein is still evident in Figure B.
R: Thank you. The object representing SpoIIIM has been removed. Updated Figure 7 is shown below.

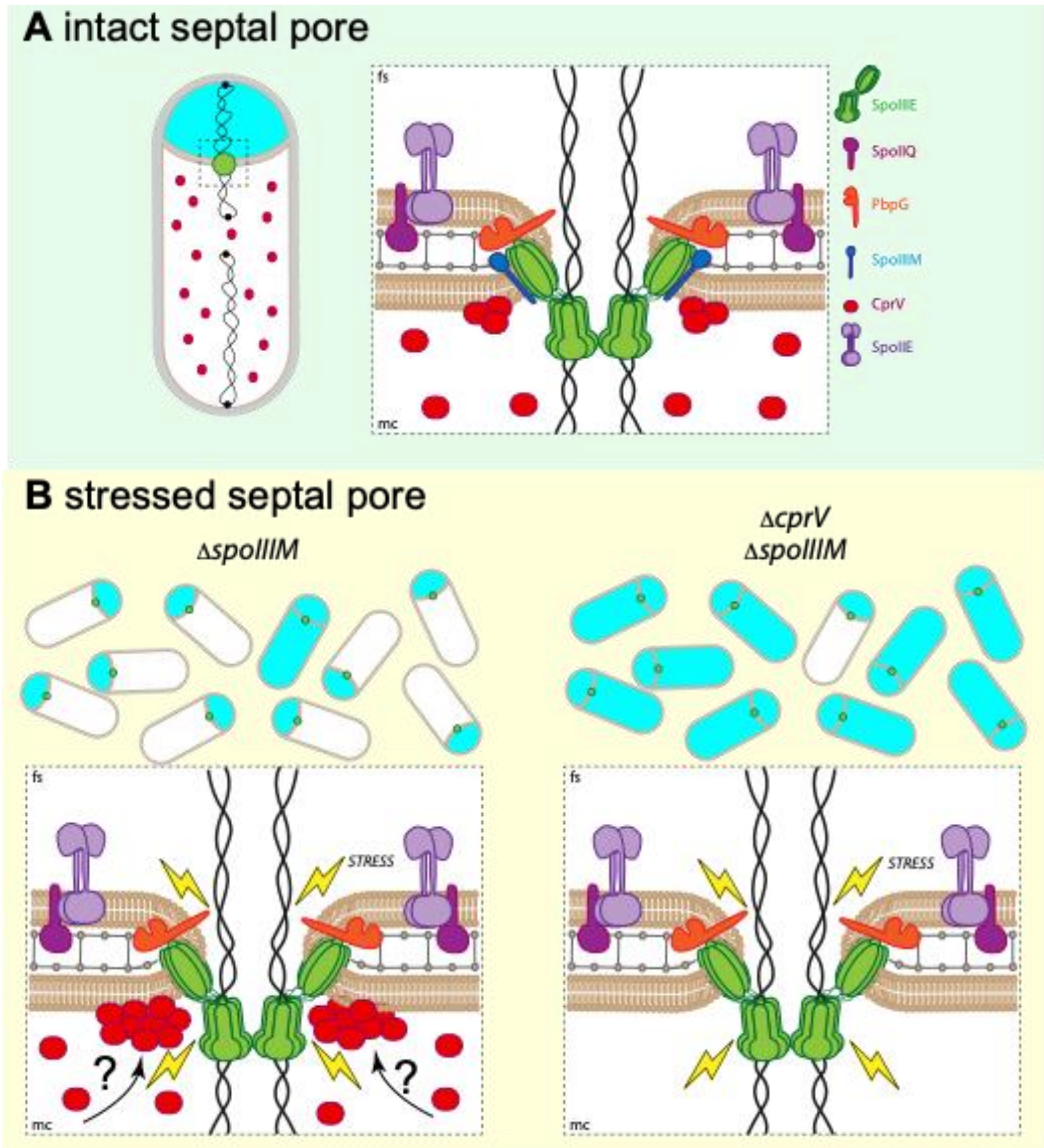

Bibliography – Ref 36 has a typo.
R: This has been amended

Reviewer #2 (Remarks to the Author):

In this article, Dehghani et al. report the characterization of a novel sporulation gene that they rename cprV. About 30 years ago, this gene was originally annotated as yteV and shown to be expressed under the control of the sporulation sigma factor SigE. Yet, its role in sporulation had remained mysterious. Here, the authors show that redundancy played a major role in masking its function. It is only when a second sporulation gene, spoIIIM (yqfZ), is missing that a clear phenotype emerges in sporulating cells. Dehghani et al. performed Tn-seq experiments to identify cprV in

the sensitized spoIIIM deletion mutant background. They performed several assays to show that compartmentalization, septal integrity, chromosome translocation (and retention in the forespore) are impaired in a spoIIIM cprV double mutant and even more drastically in a spoIIIM cprV pbpG triple mutant. In addition, they show that the DNA translocase SpoIIIE fused to GFP persists longer than in the wild type strain in the absence of cprV. Recruitment of CprV to the septal pores appears to be dependent on SpoIIIE, although probably not by direct interaction. They also show that based on similarity to the Alba family of DNA/RNA binding proteins, CprV likely forms a hexamer and identify amino acids necessary for multimerization. There are still a few minor gaps in the model regarding the recruitment of CprV to the septal pore, but on the whole the model presented in Fig.7 is supported by solid experimental evidence.
I only have a few minor suggestions listed below.

R: We thank the reviewer for their positive view of our work and appreciate their time during peer review.

Abstract: Add the original gene names (yteV, cse60) and the definition of the acronym in the abstract.

R: While we appreciate the reviewer's suggestion, adding this additional text would bring the abstract to over 200 words, which exceeds the limit permitted by the journal. Thus, we have retained the abstract. The original name is stated in the introduction, as well as the definition of the acronym, as per the original submission.

I agree that it is probably a good idea to rename the gene, because yteV is too cryptic; however, I don't completely follow the logic of calling this gene cprV (why V?). Also, previously, when the same group identified YqfZ as another compartmentalization protein, they renamed it SpoIIIM. Wouldn't it make more sense to rename YteV as SpoIIIN? Or SpoIIIM as CprZ?

R: We did consider SpoIIIN; however, based on previous experiences renaming other sporulation genes, SpoIIIN could create confusion with SpoIIIM, particularly as they appear multiple times throughout the manuscript. Hence, we chose to rename YteV as CprV. We kept the V to allude to the original name.

l.122-3 "and where the highly conserved SpoIIE-SpoIIQ interaction is abolished,". This concept requires some introduction as SpoIIE was not mentioned in the introduction and can easily be confused with SpoIIIE by readers who are not experts in the sporulation field.

R: We have elaborated on the role of SpoIIE to avoid confusion. The text included is as follows:

Page 4, Line 12: "Furthermore, in cells lacking any of these factors, and the highly conserved interaction between the phosphatase SpoIIE (which facilitates polar division and controls σ^F activation) and SpoIIQ in forespore, the septal membrane retracts[30,31]".

l.181-3 "smaller forespores or forespores with irregular membranes (Fig. 1D)." The

image shown is a bit small and too crowded to unambiguously visualize this. Maybe show a few zoomed in examples in the supplement.

R: We have added panel, Figure S1D, to show specific examples of forespores and help the reader compare forespores in the WT and mutant strains.

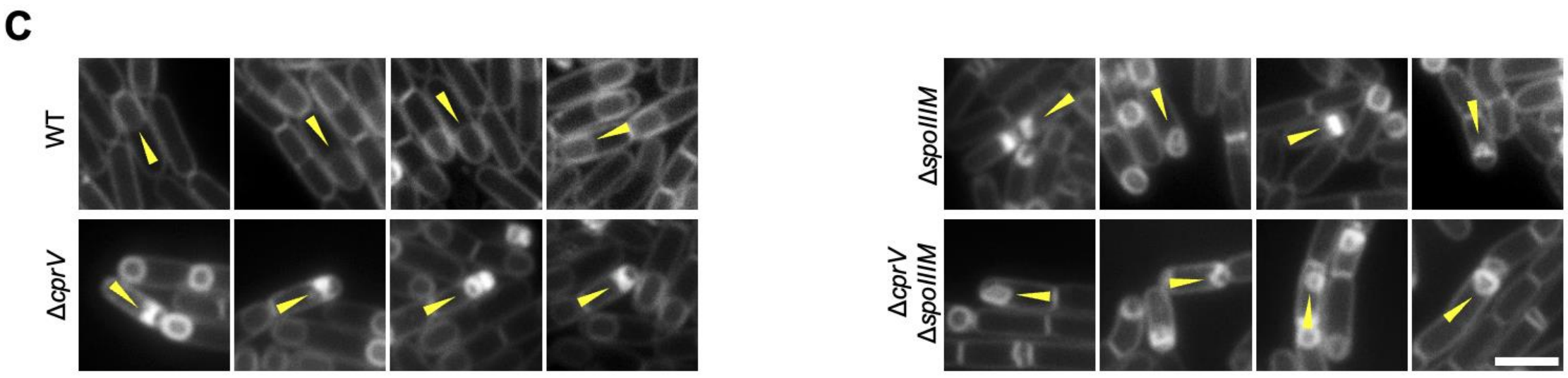

l. 218-219 “although to a lesser extent that SpoIIIM”. “than” not “that”
Fig. 4A and B, I found the legend on the y axis to be a bit confusing. It is supposed to be a comparison of the double mutant to the triple mutant, but I originally read it as a comparison of a spoIIIM deletion to cprV pbpG double mutant.

R: We have replaced “that” with “than”. Regarding Figure 4A and 4B, the comparison here is between the ***spoIIIM pbpG*** double mutant and ***spoIIIM pbpG crpV*** triple mutant, to explicitly show the effect of ***cprV.*** The legend is presented in the simplest format possible, as a means to avoid repetition of gene names and cluttering of text in the X-axis.

Reviewer #3 (Remarks to the Author):

In Bacillus subtilis, DNA translocation during sporulation occurs through a specialized septal pore that forms during polar septation over the partially segregated forespore chromosome. This process is tightly coordinated with septal peptidoglycan (PG) remodeling to preserve compartmentalization between the mother cell and forespore. Central to this coordination is a complex comprising the DNA translocase SpoIIIE, the forespore-produced PG synthase PbpG, and the mother-cell protein SpoIIIM, which together maintain septal pore stability by balancing PG synthesis and hydrolysis. This balance ensures that the pore remains sufficiently large to allow DNA passage while remaining constrained enough to prevent cytoplasmic leakage.
Loss of SpoIIIM and PbpG significantly affects compartmentalization but not spore formation. In contrast, in the absence of SpoIIIE, all cells become miscompartmentalized and no spores are formed. These observations suggest that additional SpoIIIE-dependent factors contribute to septal pore stability and compartmentalization.

This study identifies CprV as a new protein involved in maintaining gene-expression compartmentalization during sporulation in response to septal pore stability defects. Although the cprV single mutant displays only mild defects in compartmentalization and chromosome translocation, combining cprV deletion with mutations in spoIIIM and/or pbpG exacerbates these phenotypes. These double and triple mutants show reduced formation of heat-resistant spores, altered SpoIIIE localization dynamics, defects in chromosome translocation into the forespore, and increased

miscompartmentalization of gene expression. CprV is a small 60 amino acid protein with predicted structural similarity to Alba proteins. The authors show that CprV can self-interact, and in silico predictions suggest that it may form a hexamer. Substitutions in residues located at the predicted monomers interface disrupt CprV self-interaction and impair its function in vivo.

Overall, the manuscript is clearly and convincingly presented. The experiments are rigorous and, in general, well interpreted, and the study represents an important contribution to the field. I have only a few suggestions that may help improve the manuscript.

R: We thank the reviewer for their positive assessment of our work and appreciate their time during peer review.

Main comment: It remains somewhat difficult to distinguish miscompartmentalization caused by cytoplasmic leakage from ectopic activation of compartment-specific sigma factors. The cprV single mutant also shows some degree of miscompartmentalization; does this imply that septal pore stability is compromised even in this background? If so, could this be directly assessed, for example by electron microscopy or another approach capable of visualizing septal pore architecture? This point seems particularly relevant because, unlike the suppression observed upon deletion of spoIID and spoIIP in other septal pore stability mutants, loss of PG hydrolysis in the cprV mutant appears to suppress chromosome translocation defects but not the miscompartmentalization of gene expression. An alternative possibility is that CprV may have a more direct role in regulating compartment-specific gene expression. Given its predicted structural similarity to Alba proteins, despite its acidic nature, it would be useful to discuss whether CprV could influence gene expression independently of, or in addition to, effects on septal pore stability.

R: Examination of the septal pore architecture is something we have considered. However, random sectioning of the cells for electron microscopy limits the likelihood of finding the septal pore within any of the sections generated. Thus, a more targeted approach would be required to directly image the septal pore. This is something we hope to address in future work exploring the septal pore.

Regarding a more direct role for CprV in regulating compartment-specific gene expression, we consider this unlikely, as CprV does not appear to bind nucleic acids. Instead, based on our data, we favour the model that CprV supports septal pore stability, even when septal PG hydrolysis is blocked. Our data show that blocking septal peptidoglycan hydrolysis restores compartmentalisation to all cells lacking CprV. However, when septal peptidoglycan hydrolysis is blocked and if CprV is absent in cells lacking PbpG, SpoIIIM and PbpG, or SpoIIIM, PbpG and SpoIIQ, a fraction of cells remains miscompartmentalised. This suggests that the septal pore remains “leaky” in specific mutant backgrounds lacking CprV, but not in the absence of CprV alone. Thus, our interpretation is that CprV ensures the septal pore remains stable, if other septal stabilization factors are missing.

In the context of our experiments and what is known about σ^F activation, it is difficult to envisage a scenario where CprV prevents ectopic activation of σ^F, particularly since SigF activation is controlled by SpoIIE in the forespore and CprV is transcribed in the mother cell. If CprV was somehow involved in preventing σ^F activation in the mother cell, then we would expect to observe miscompartmentalisation of σ^F in the absence of CprV when PG hydrolysis is blocked; this is not the case, as miscompartmentalisation in the absence of CprV only occurs in cells undergoing engulfment (active PG hydrolysis). Thus, it is difficult to envisage a model where CprV prevents ectopic activation of σ^F, only during engulfment or when PG hydrolysis is blocked in specific mutant backgrounds, as stated above. Nonetheless, we have alluded to this possibility in the discussion, pointing out more directly that blocking septal peptidoglycan hydrolysis in the absence of CprV, in certain mutant backgrounds, is not sufficient to fully restore compartmentalisation. The text included is as follows:

Page 17; Line 10: "The additional stability CprV provides to the septal pore was also apparent when septal PG hydrolysis was blocked in cells lacking PbpG, SpoIIIM and PbpG, or SpoIIIM, PbpG and SpoIIQ. In these mutants CprV appears to be essential for maintaining compartmentalisation, as blocking septal PG hydrolysis was insufficient to restore compartmentalisation to all cells (Fig. S2A&B). While these data suggest that CprV functions to safeguard septal pore stability, it is formerly possible that CprV functions to prevent ectopic σ^F activation in specific mutant backgrounds. However, since σ^F activation is controlled by SpoIIE in the forespore and CprV is transcribed in the mother cell, we do not favour this model."

Line 78: consider revising "σF, σE, σG and σK" to superscript throughout the manuscript

R: This has now been revised.

Lines 489–494: It would be important to confirm that the CprVV4A allele accumulates to levels comparable to wild-type CprV in Bacillus subtilis cells. Without this control, the conclusion that loss of function is specifically due to impaired multimerization should be made with caution, as the V4A substitution could also affect protein stability, expression, or accumulation.

R: This is something we attempted before submitting the manuscript. As we do not have a CprV antibody, the V4A mutation was made in the context of a functional CprV-His6 fusion, so we could detect CprV using an anti-His antibody. Unfortunately, for reasons that remain unclear we were unable to detect CprV-His6 using a His antibody. Based on this, and following the reviewer's suggestion, we make this explicit in the main text and caution the conclusion around this data. The modified section of text included is as follows:

Page 14, Line 12: "To test the functional significance of the CprV-CprV interaction in vivo, we introduced the CprV^{V4A} mutant, in the context of a functional C-terminal, hexahistidine-tagged version of CprV (CprV-His6) (Fig. 6E), at an ectopic locus in ΔcprV and ΔcprV ΔspoIIIM mutant strain backgrounds. We then examined the ability of the CprV^{V4A} mutant to complement sporulation and compartmentalization defects. CprV^{V4A} produced sporulation and compartmentalization defects that were identical

to a ΔcprV mutant and failed to complement CprV in both backgrounds (Fig. 6D-F). For reasons unknown, we were unable to detect CprV-His6 with His6 antibodies by immunoblot and confirm the levels of CprV^{V4A}. Thus, we tentatively conclude that multimerisation is required for CprV's role in compartmentalization."

Page 14, Line 22: "Taken together, these data suggest that CprV may multimerize in vivo and that multimerisation may be ***required for CprV's role in compartmentalization."***

Page 16, Line 35: "…and this multimerisation may be required for CprV function, including compartmentalization (Fig. 6D-F)."

While revising the manuscript, we realized that the plasmid used to introduce the CprV V4A -His6 construct mutant was not listed in the supplementary information. This has now been included. Furthermore, we also include data demonstrating that CprV-His6 is functional – this was added to Fig. 6E. The updated Fig. 6E is shown below.

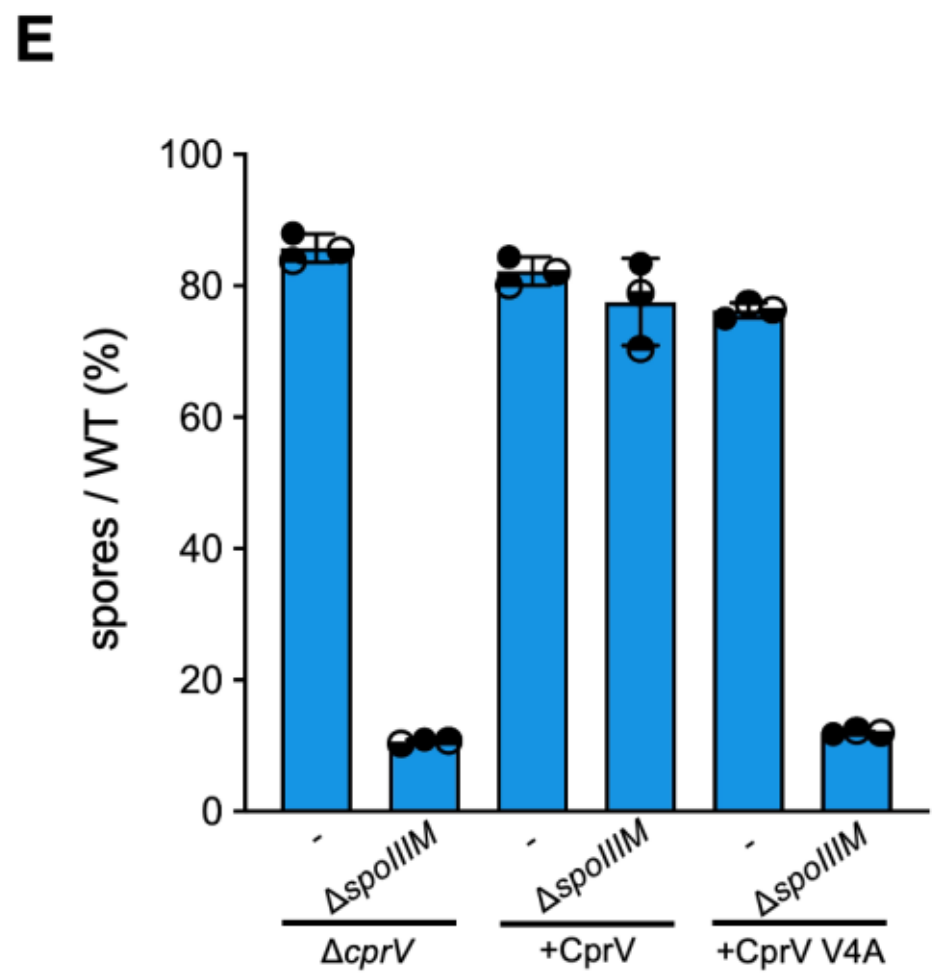

Figure 1 legend: "(refs)" is still present in the figure legend and should be replaced with the appropriate references.

R: This has now been replaced.

Dehghani et al – Response to Reviewer Comments (in blue)

Reviewer #2 (Remarks to the Author):

I believe that the authors have addressed all the comments satisfactorily. There is just one response that I disagree with. I still think that it would be helpful to have the original name of the gene (yteV) mentioned in the abstract. I know from personal experience that literature searches in various databases are greatly facilitated when synonyms are clearly spelled out. I also don't think that word count is a valid argument to deny this request as it only adds one word to the abstract.

R: We were under the impression that there was a word limit – hence we did not include it. Following the editor's recommendation, we have now added YteV in the abstract. The abstract now reads, Page 2, Line 9:

"Here, we identify CprV (YteV) as a safeguarding factor that maintains compartmentalization in response to septal pore stability defects"

Reviewer #3 (Remarks to the Author):

The original manuscript was excellent. Only minor revisions were requested, and the authors have in same way addressed them.

R: Thank you. We are glad to have addressed the reviewer's concerns.
