# Supplementary material for: A safeguarding factor monitors septal pore stability to secure compartmentalization during sporulation in Bacillus subtilis
Source: Commun Biol. 2026 Jul 15;9:1271. doi: 10.1038/s42003-026-10705-y (PMC13627676; doi:10.1038/s42003-026-10705-y)
Supplement: Supplementary file 2 [file 42003_2026_10705_MOESM2_ESM.pdf]

SUPPLEMENTARY INFORMATION

A Safeguarding Factor Monitors Septal Pore Stability to Secure Compartmentalization during Sporulation in *Bacillus subtilis*

Behzad Dehghani[1], Chris L.B. Graham[1], Helena Chan[2], Najwa Taib[3] & Christopher D.A. Rodrigues[1]

[1]School of Life Sciences, University of Warwick, Coventry, UK

[2]Australian Institute for Microbiology & Infection, University of Technology Sydney, Australia

[3] Hub Bioinformatics & Biostatistics, Department of Computational Biology, Institut Pasteur, Paris, France

Keywords: compartmentalization, sporulation, chromosome translocation, SpoIIIE

*corresponding-author

email: christopher.rodrigues@warwick.ac.uk

Tel: +44 (0) 24 761 50058

A

No	Gene	*p*-value	Tn. Fold change	No	Gene	*P*-value	Tn. Fold change
1	*spoIIIM*	-	-	9	*yddC*	0.0259	21
2	*pbpG*	0.00008	6.8	10	*fadE*	0.01	2.53
3	*yugM*	0.01682	3.6	11	*yopT*	0.0045	3.3
4	*yteV (cprV)*	0.04198	5.9	12	*mraW*	0.0089	11.11
5	*sspC*	0.02776	4.1	13	*ylmg*	0.0413	3.55
6	*gltC*	0.00301	5.2	14	*etfB*	0.0289	7.01
7	*yrdA*	0.01352	5.2	15	*yqcK*	0.0308	7.87
8	*clsA*	0.04878	2.8	16	*yubB*	0.0194	4.09

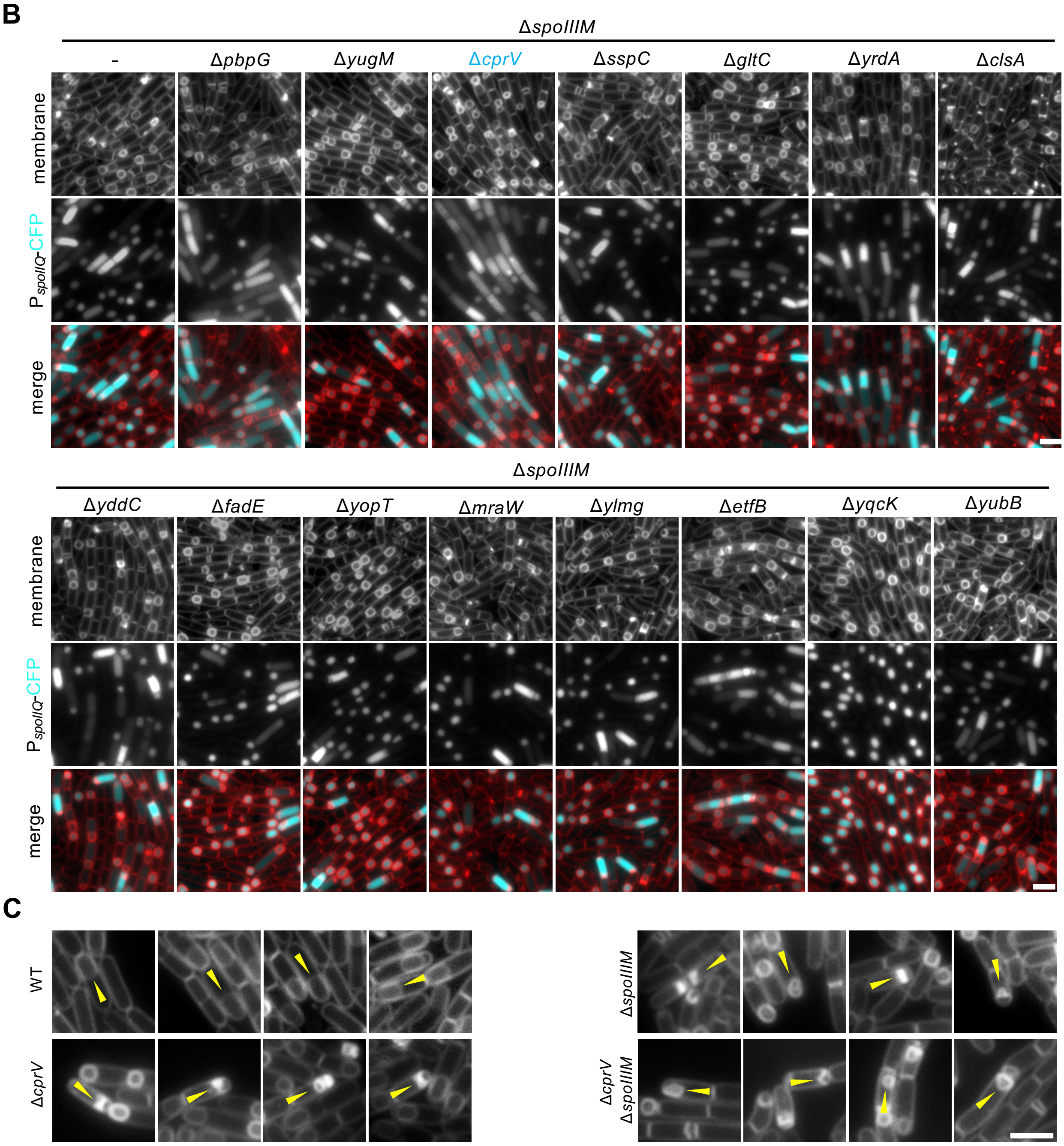

Figure S1. Top Tn-seq hits and cytologically screening of mutants that enhance the compartmentalization defect of the Δ*spoIIIM* mutant defect. (A) List of the top 15 Tn-seq hits, including *p*-value and transposon insertion fold-change, in the Δ*spoIIIM* mutant relative to the WT strain. **(B)** Representative images of double mutant compartmentalization defects compared to the Δ*spoIIIM* mutant. The Δ*cprV* Δ*spoIIIM* double mutant exhibits an increased miscompartmentalization phenotype compared with Δ*spoIIIM*. **(C)** Enlarged representative forespore images highlighting membrane defects in Δ*cprV*, Δ*spoIIIM*, and Δ*cprV* Δ*spoIIIM* strains. Scale bar, 2 µm.

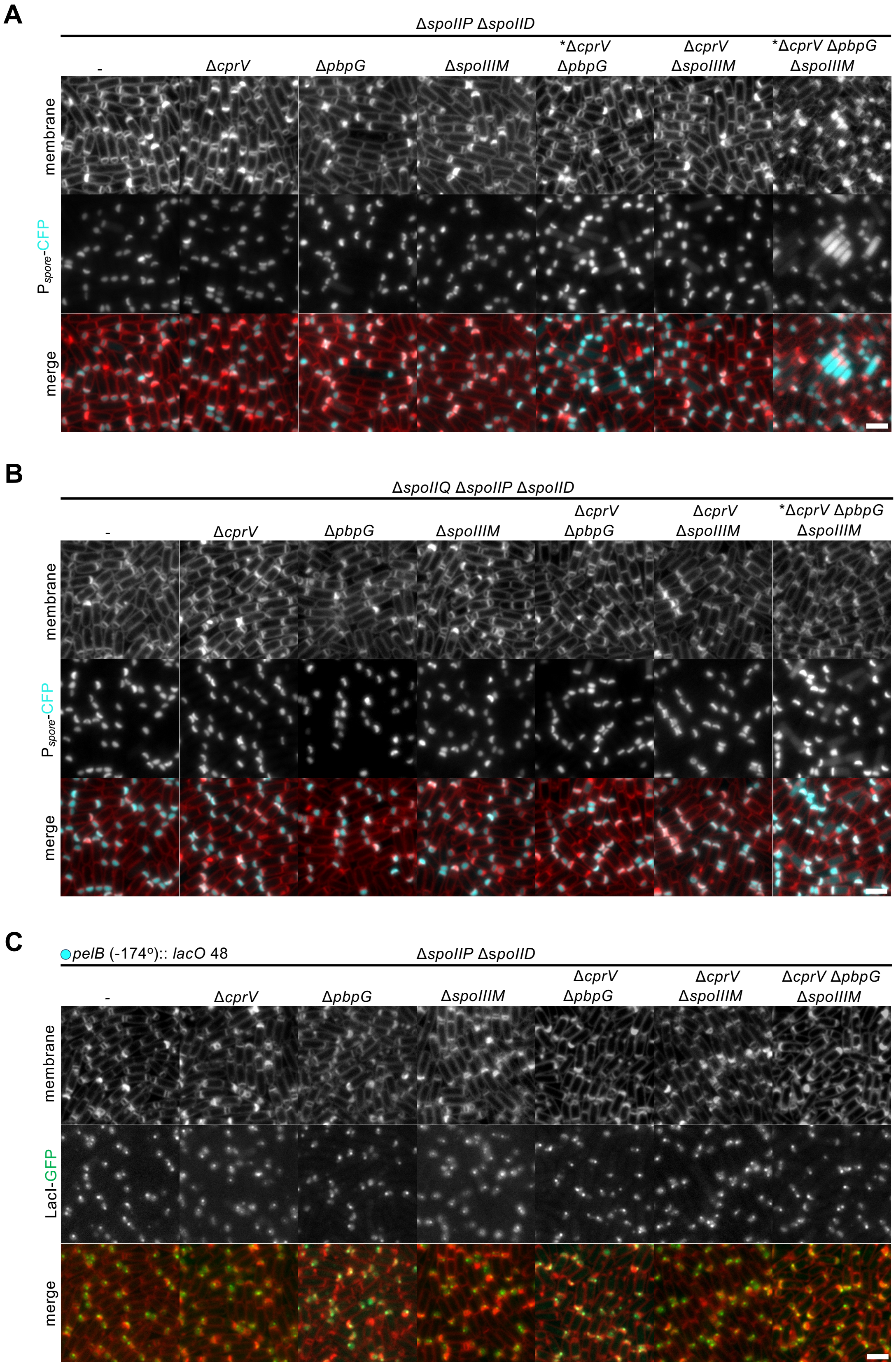

Figure S2. Suppression of miscompartmentalization and chromosome translocation defects in the Δ*spoIID* Δ*spoIIP* background. (A) Representative images showing suppression of miscompartmentalization defects in the Δ*spoIID* Δ*spoIIP* background at T3, in WT, Δ*cprV,* Δ*spoIIIM*, Δ*pbpG*, Δ*spoIIIM* Δ*cprV,* Δ*pbpG* Δ*cprV* and Δ*cprV spoIIIM* Δ*pbpG* mutant strains. **(B)** Representative images showing suppression of miscompartmentalization defects in the Δ*spoIID* Δ*spoIIP* background at T3, in Δ*spoIIQ*, Δ*spoIIQ* Δ*cprV,* Δ*spoIIQ* Δ*spoIIIM*, Δ*spoIIQ* Δ*pbpG*, Δ*spoIIQ* Δ*spoIIIM* Δ*cprV,* Δ*spoIIQ* Δ*pbpG* Δ*cprV* and Δ*spoIIQ* Δ*cprV spoIIIM* Δ*pbpG* backgrounds.***Note**: In three mutant backgrounds, partial suppression of miscompartmentalization was observed: 26% (±2.1 SD) in the Δ*pbpG* Δ*cprV*, 33% (±3.95 SD) in Δ*cprV spoIIIM* Δ*pbpG*, and 25% (±1.25 SD) in the Δ*spoIIQ* Δ*cprV spoIIIM* Δ*pbpG*. **(C)** Representative images showing suppression of chromosome translocation defects in the Δ*spoIID* Δ*spoIIP* at T3, in WT, Δ*cprV,* Δ*spoIIIM*, Δ*pbpG*, Δ*spoIIIM* Δ*cprV,* Δ*pbpG* Δ*cprV* and Δ*cprV spoIIIM* Δ*pbpG* using *lacO48* sites inserted at the *pelB* locus. Scale bar: 2 μm.

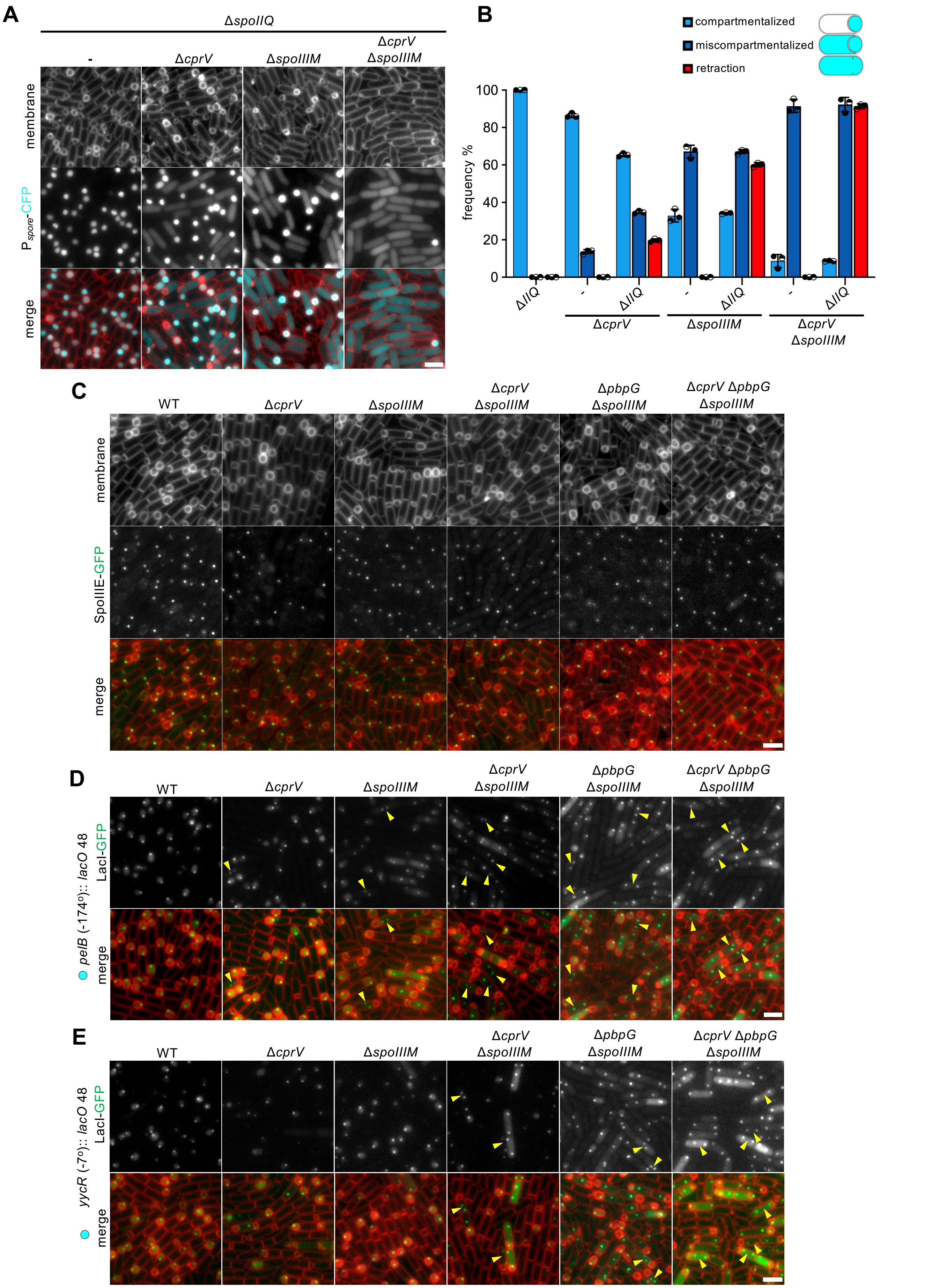

Figure S3. Septal retraction, SpoIIIE-GFP localization and chromosome translocation defects in various mutant backgrounds. (A) Representative images of septal retraction in WT, Δ*cprV*, Δ*spoIIIM*, and Δ*cprV* Δ*spoIIIM* in the Δ*spoIIQ* background at T3. Scale bar, 2 µm. **(B)** Average frequency (± SD of 3 biological replicates) of cells exhibiting septal retraction in WT, Δ*cprV*, Δ*spoIIIM*, and Δ*cprV* Δ*spoIIIM* in the Δ*spoIIQ* background at T3 (n>250 per time point, per strain, per replicate). **(C)** Representative images of SpoIIIE-GFP localization in WT, Δ*cprV*, Δ*spoIIIM*, Δ*cprV* Δ*spoIIIM,* Δ*pbpG* Δ*spoIIIM* and Δ*cprV spoIIIM* Δ*pbpG* mutant strains at T2. Scale bar, 2 µm. **(D)** Representative images showing LacI-GFP foci in WT, Δ*cprV*, Δ*spoIIIM*, Δ*cprV* Δ*spoIIIM,* Δ*pbpG* Δ*spoIIIM* and Δ*cprV spoIIIM* Δ*pbpG* mutant strains at T2 carrying *lacO48* at the *pelB locus*. Scale bar, 2 µm. **(E)** Representative images showing LacI-GFP foci at T2 in the same mutant strains shown in (D) carrying *lacO48* at the *yycR* locus. Scale bar, 2 µm.

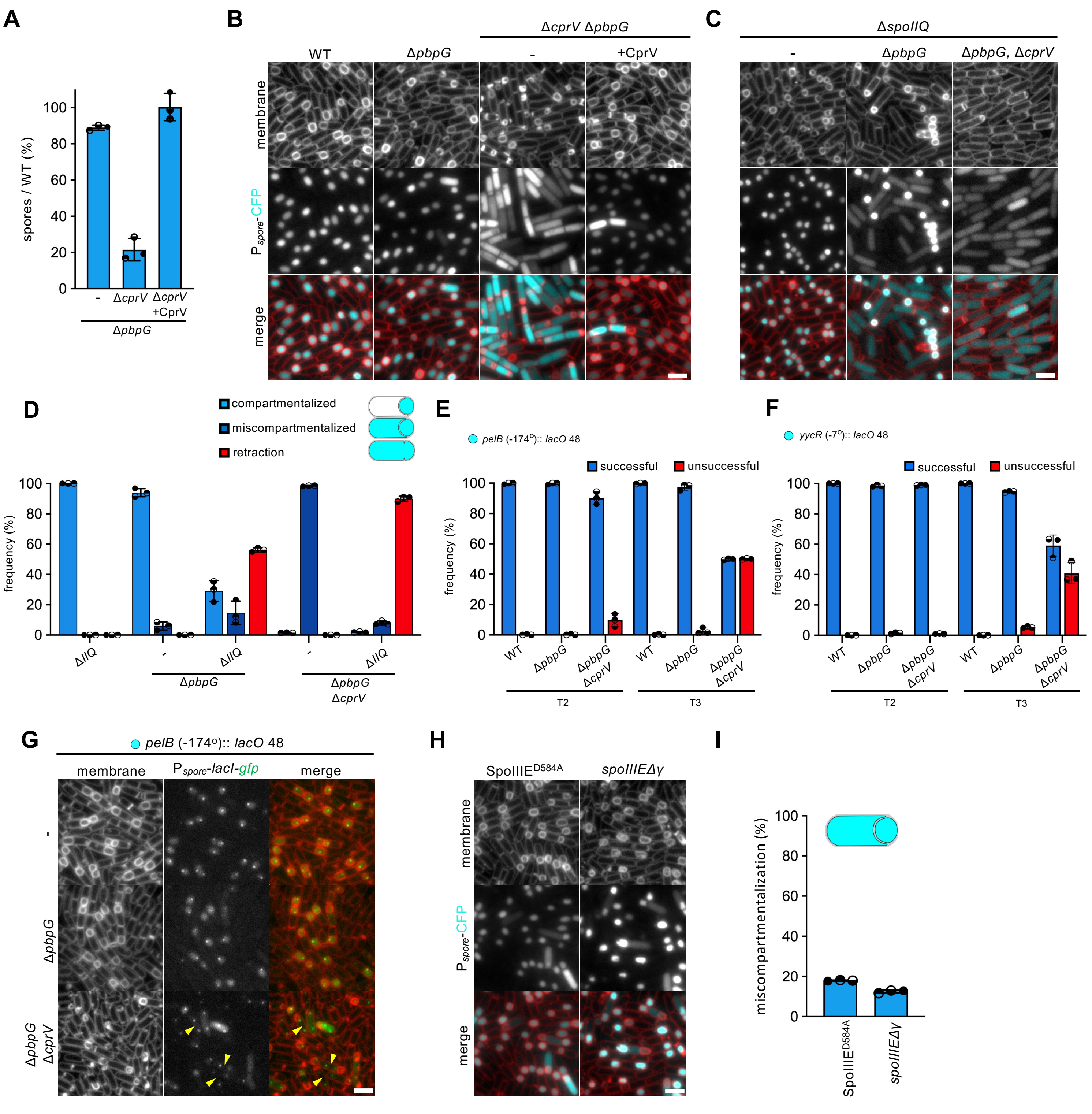

Figure S4. **Evidence that CprV and PbpG act synergistically to maintain septal integrity and efficient chromosome translocation during sporulation and compartmentalization frequency in SpoIIIE mutants. (A)** Average sporulation efficiency (% ± SD, n = 3) of Δ*pbpG*, Δ*cprV*, Δ*cprV* Δ*pbpG*, and the corresponding *cprV*-complemented Δ*cprV* Δ*pbpG* strain. **(B)** Representative images showing miscompartmentalization in WT, Δ*pbpG*, Δ*cprV* Δ*pbpG*, and the *cprV*-complemented strain. **(C)** Septal retraction in Δ*pbpG* and Δ*cprV* Δ*pbpG* strains in a Δ*spoIIQ* background at T3. Scale bar, 2 µm. **(D)** Average frequency (± SD from three biological replicates) of cells exhibiting miscompartmentalization or septal retraction at T3 in WT, Δ*pbpG*, Δ*cprV* Δ*pbpG*, and the *cprV*-complemented strain (n > 200 per time point, per strain, per replicate). **(E & F)** Average frequency (± SD from three biological replicates) of cells exhibiting successful chromosome translocation or unsuccessful translocation at T2 and T3, in WT, Δ*pbpG*, and Δ*cprV* Δ*pbpG* strains with *lacO48* integrated at either the *pelB* locus **(E)** or the *yycR* locus **(F)** (n > 250 per time point, per strain, per replicate). **(G)** Representative fluorescence images showing LacI-GFP foci at T3 in WT, Δ*pbpG*, Δ*cprV*, and Δ*cprV* Δ*pbpG* strains with *lacO48* inserted at the *pelB* locus. Yellow arrows represent examples of unsuccessful chromosome translocation in each background. Scale bar, 2 µm. **(H)** Representative images showing miscompartmentalization in SpoIIIE^{D584A} and *spoIIIEΔγ* strains. Scale bar, 2 µm. **(I)** Average frequency (± SD from three biological replicates) of cells exhibiting miscompartmentalization in SpoIIIE^{D584A} and *spoIIIEΔγ.* (n > 250 per strain, per replicate).

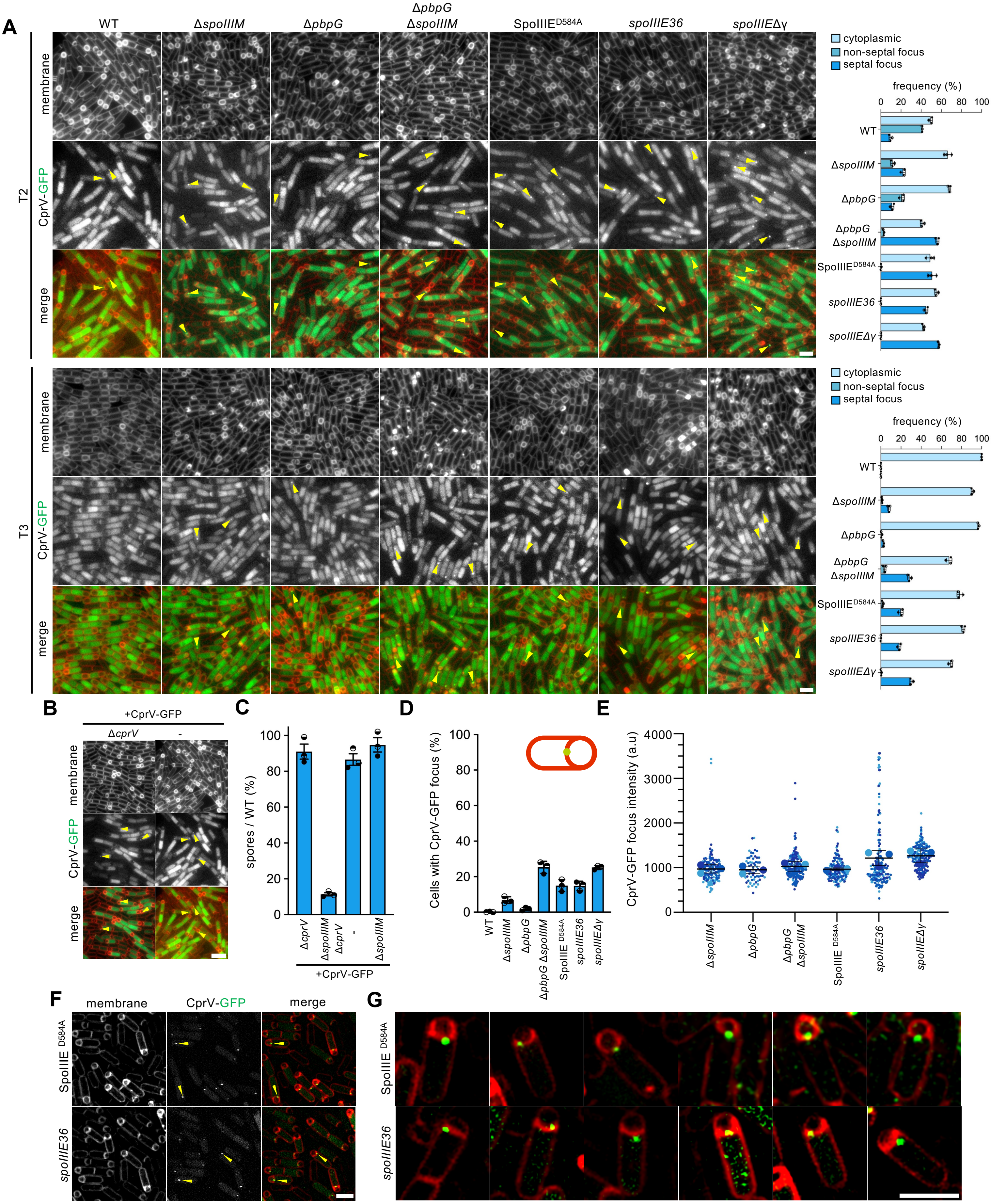

Figure S5. CprV-GFP localization during engulfment. (A) Representative images and quantification of CprV-GFP localization in WT, Δ*spoIIIM*, Δ*pbpG*, Δ*pbpG* Δ*spoIIIM*, SpoIIIE^{D584A}, *spoIIIE36* and *spoIIIEΔγ* strains at T2 and T3. Scale bar, 2 µm. Average frequency (± SD from three biological replicates) of cells containing a CprV-GFP septal focus, non-septal focus or cytoplasmic CprV-GFP only, at T2 and T3. **(B)** Representative images of CprV-GFP localization when the only copy of CprV is CprV-GFP and merodiploid strain (when the CprV-GFP is present alongside native *cprV* allele). **(C)** Average sporulation efficiency (± SD, n=3) of strains with CprV-GFP as the sole source of CprV or CprV-GFP in merodiploid background. **(D)** Average frequency (± SD from three biological replicates) of cells containing a CprV-GFP septal focus in the same strains shown in (A) at T3. **(E)** Quantification of CprV-GFP septal focus fluorescence signal intensity (average ± STDEVP; n > 200 per strain, per time point) in the indicated strains at T3. **(F)** Super-resolution images of CprV-GFP localization in *spoIIIE*^{D584A} and *spoIIIE36* strains at T2. Scale bar, 2 µm. Yellow arrowheads indicate a septal focus. **(G)** Enlarged representative images showing septal localization of CprV-GFP in SpoIIIE^{D584A} and *spoIIIE36* strains. Scale bar, 2 µm.

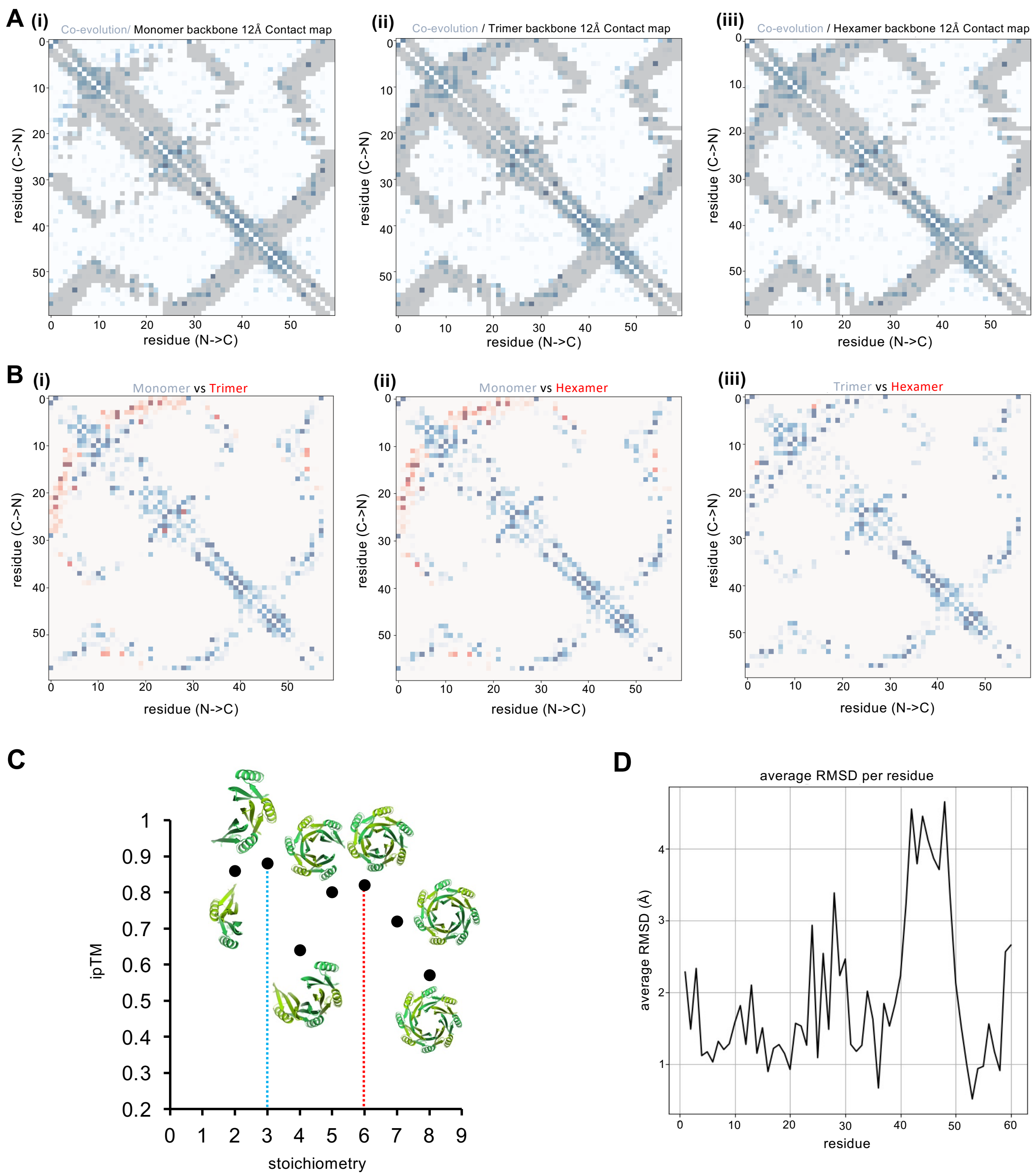

Figure S6. CprV multimerization predicted by Alphafold3 and co-evolutionary analysis. (A) Co-evolution matrix of CprV after 100 iterations. Blue - Higher vmax values, vmin =0.5, vmax =3.0. Grey – 12Å distance backbone contact map of monomer (i), trimer (ii) and hexamer (iii), respectively. **(B)** Co-evolution matrix showing residue contact maps predicted by AlphaFold3, monomer vs trimer (i), monomer vs hexamer (ii) and trimer vs hexamer (iii). Red indicates co-evolutionary interactions that are exclusive to the trimer or hexamer, when compared to the monomer or trimer. **(C)** AlphaFold3 multimer stoichiometry plotted against ipTM values. Hexamer and/or trimer produce higher ipTM scores. **(D)** RMSD per residues plot of hexamer during full atom GROMACs simulation for 100 ns at 200 mM NaCl 37ºC.

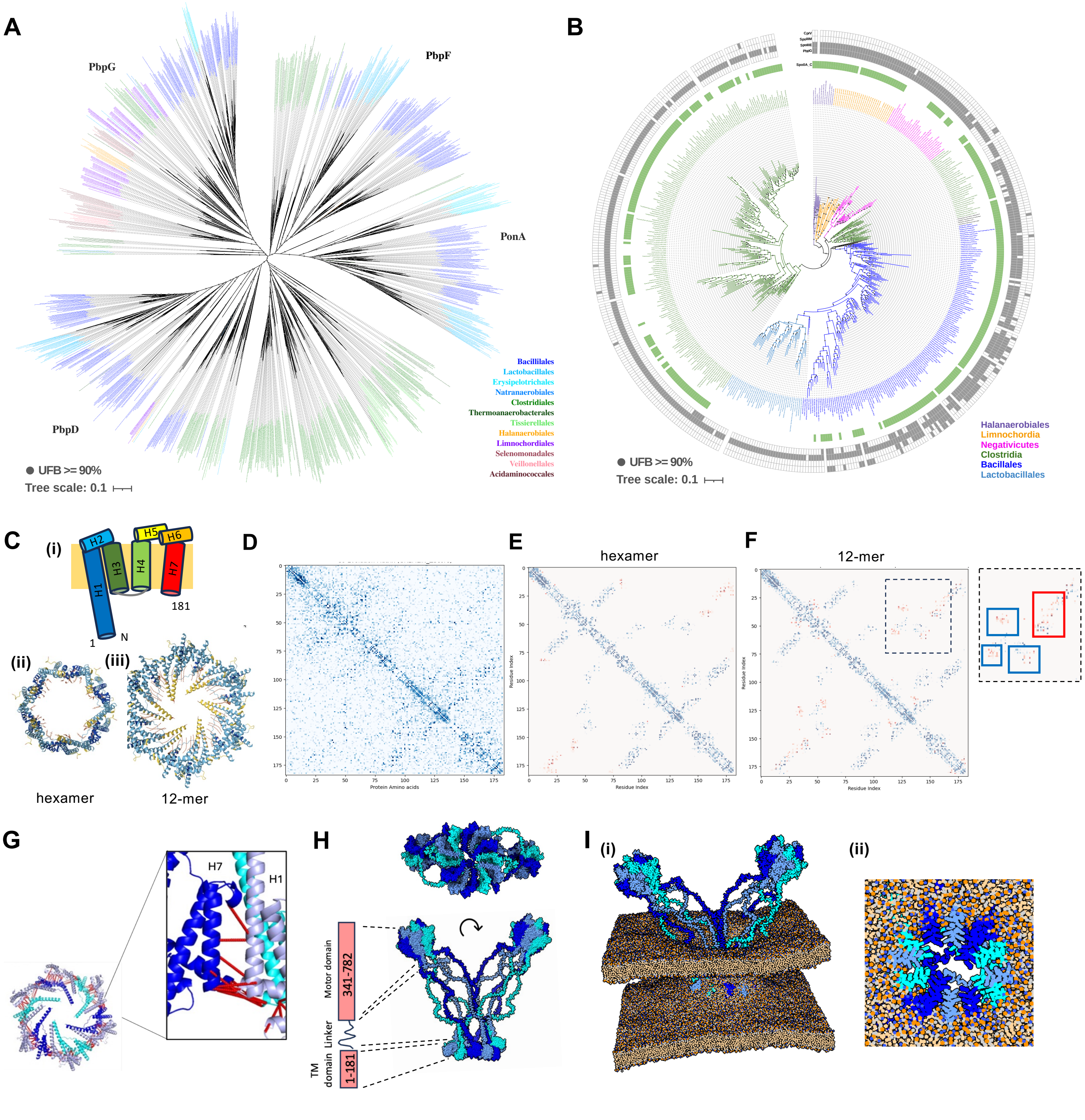

Figure S7: Phylogenetic distribution of SpoIIIE, SpoIIM, PbpG and CprV on a reference phylogeny of *Firmicutes* and modelling of SpoIIIE complex at toroidal membrane pore. (A) Maximum likelihood phylogeny of class-A PBP protein family in the *Firmicutes,* used to define phylogeny of PbpG vs other class-A PBP. **(B)** Phylogenetic distribution of PbpG, SpoIIIE, SpoIIM, CprV (in gray) and Spo0A_C as a marker of sporulation (in green) on a maximum likelihood reference phylogeny of the *Firmicutes* based on a supermatrix containing 497 taxa with 3,776 amino acid positions and inferred with IQ-Tree using the evolutionary model LG+I+G4. Black circles correspond to ultrafast bootstrap supports higher than 90%, and the scale bar corresponds to the average number of substitutions per site. **(C)** Predicted membrane topology of SpoIIIE transmembrane domain (TMD; amino acids 1-181) (i) and cartoon model AlphaFold3 prediction of hypothetical 6-mer (ii) and 12-mer (iii) SpoIIIE TMD with oleic acid. pLDDT (blue high confidence) - (yellow low confidence) ipTM = 0.4 and ipTM = 0.29 respectively. **(D)** Co-evolution matrix of SpoIIIE TMD predicted using CoEVFolD (refs). **(E)** Co-evolution matrix of SpoIIIE TMD hexamer. Red shows co-evolution exclusively identified in the TMD hexamer compared to TMD in the monomer (blue). **(F)** Co-evolution matrix of SpoIIIE TMD 12-mer. Red shows co-evolution exclusively identified in the TMD 12-mer compared to TMD hexamer (blue). Inset boxes - red box identifies coevolution between H1-H7 interactions; Blue box identifies coevolution at turns between H2 and H7.**(G)** H2 and additional H1-H7 interactions exclusively found in SpoIIIE TMD 12-mer model. **(H)** Domain architecture of full-length SpoIIIE modelled with two hexameric motor domains joined by a multimeric TMD 12-mer. Note that the SpoIIIE TMD 12-mer is stable when simulated in a toroidal membrane pore using MemTorMD[58]. **(I)** Illustrative model demonstrating hypothetical insertion of SpoIIIE complex model in (H) at a toroidal membrane with a 5 nm pore; side view (i); close-up of the interior of pore showing arrangement of SpoIIIE TMD 12-mer domain (ii).

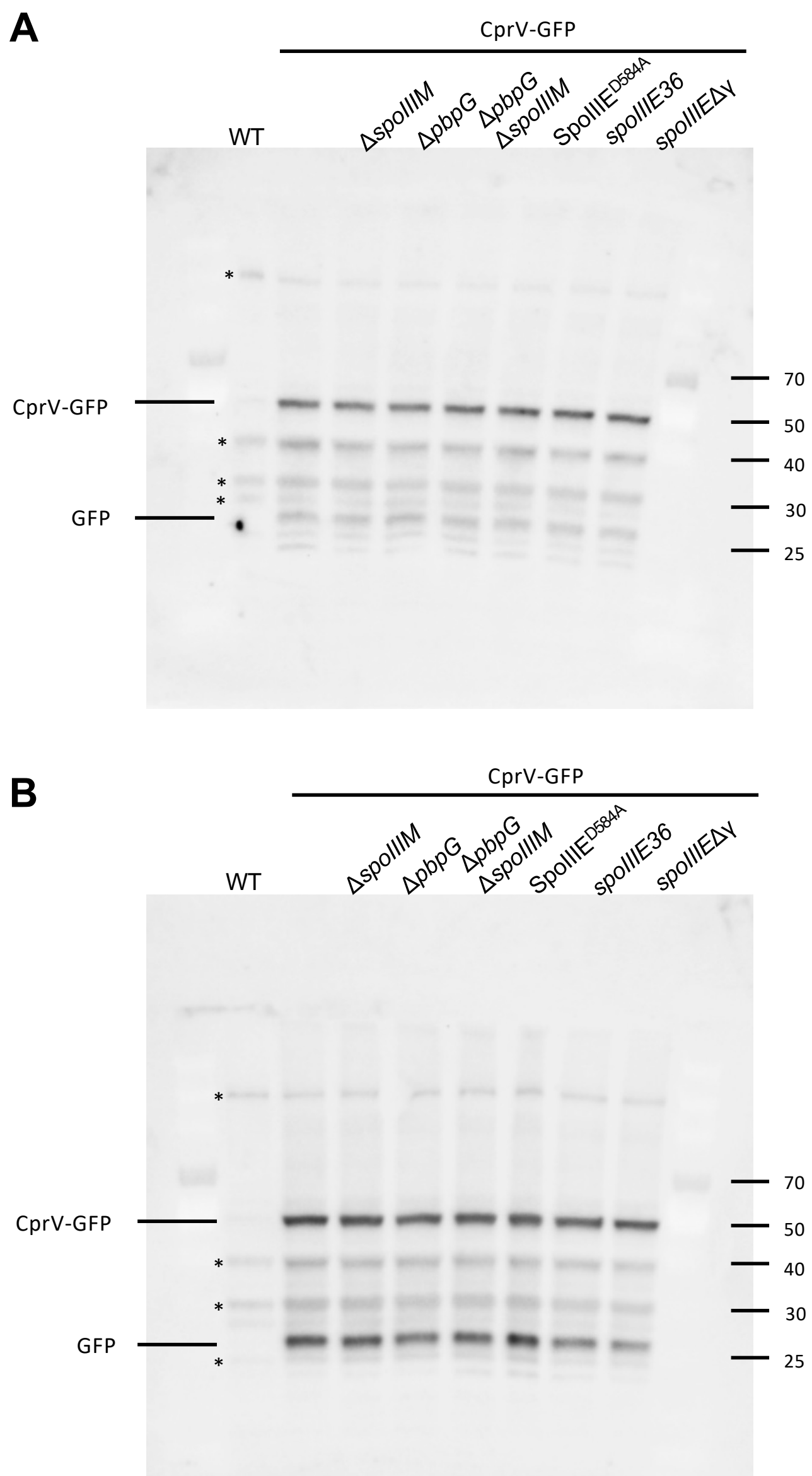

Figure S8: Uncropped immunoblots of cell lysates containing CprV-GFP, collected at T2 (A) and T3 (B). The positions of CprV-GFP and GFP are indicated to the left and position of molecular weight markers to the right. WT refers to the control strain lacking GFP. Asterisks indicate non-specific bands.

Table S1: Strains, plasmids and primers used in this study.

Bacillus subtilis strains

Name	Genotype	Source
bAT87	amyE::PspoIIQ-cfp(Bs) (cat)	1
bAT88	amyE::PspoIIQ-cfp(Bs) (cat), spoIIIM::erm	1
bAT89	amyE::PspoIIQ-cfp(Bs) (cat), pbpG::kan	1
bAT91	amyE::PspoIIQ-cfp(Bs) (cat), pbpG::kan, spoIIIM::erm	1
bAT469	amyE::PspoIIQ-cfp(Bs) (cat), spoIID::spec, spoIIP::tet	1
bAT470	amyE::PspoIIQ-cfp(Bs) (cat), spoIID::spec, spoIIP::tet, spoIIIM::lox72	1
bAT471	amyE::PspoIIQ-cfp(Bs) (cat), pbpG::lox72, spoIID::spec, spoIIP::tet,	1
bAT478	amyE::PspoIIQ-cfp(Bs) (cat), spoIIQ::erm	1
bAT479	amyE::PspoIIQ-cfp(Bs) (cat), spoIIQ::erm, spoIIIM::lox72	1
bAT480	amyE::PspoIIQ-cfp(Bs) (cat), pbpG::lox72, spoIIQ::erm	1
bAT481	amyE::PspoIIQ-cfp(Bs) (cat), pbpG::lox72, spoIIQ::erm, spoIIIM::lox72	1
bAT490	amyE::PspoIIQ-cfp(Bs) (cat), spoIID::spec, spoIIP::tet, spoIIQ::erm	1
bAT491	amyE::PspoIIQ-cfp(Bs) (cat), spoIID::spec, spoIIP::tet, spoIIQ::erm, spoIIIM::lox72	1
bAT492	amyE::PspoIIQ-cfp(Bs) (cat), pbpG::lox72, spoIID::spec, spoIIP::tet, spoIIQ::erm	1
bAT588	spoIIIE-gfp (spec)	1
bAT589	spoIIIE-gfp (spec), spoIIIM:: lox72	1
bAT591	pbpG:: lox72, spoIIIE-gfp (spec), spoIIIM::lox72	1
bAT627	amyE::PspoIIQ-spoIVFRBS-lacI-gfp (spec), spoIIIM::lox72, yycR::lacO48 (cat)	1
bAT628	amyE::PspoIIQ-spoIVFRBS-lacI-gfp (spec), spoIIIM::lox72, yycR::lacO48 (cat)	1
bAT629	amyE::PspoIIQ-spoIVFRBS-lacI-gfp (spec), pbpG::lox72, spoIIIM::lox72, yycR::lacO48 (cat)	1
bAT630	amyE::PspoIIQ-spoIVFRBS-lacI-gfp (spec), pelB::lacO48 (kan), spoIIIM::lox72	1
bAT631	amyE::PspoIIQ-spoIVFRBS-lacI-gfp (spec), pbpG::lox72, pelB::lacO48 (kan)	1
bAT632	amyE::PspoIIQ-spoIVFRBS-lacI-gfp (spec), pbpG::lox72, pelB::lacO48 (kan), spoIIIM::lox72	1
bAT646	amyE::PspoIIQ-spoIVFRBS-lacI-gfp (spec), pelB::lacO48 (kan)	1
bAT648	amyE::PspoIIQ-spoIVFRBS-lacI-gfp (spec), yycR::lacO48 (cat)	1
bAT656	amyE::PspoIIQ-cfp(Bs) (cat), spoIIIE::neo, ycgO::spoIIIEΔγ (spec)	
bAT697	amyE::PspoIIQ-spoIVFRBS-lacI-gfp (spec), pelB::lacO48 (kan), spoIID::cat, spoIIP::tet	1
bAT698	amyE::PspoIIQ-spoIVFRBS-lacI-gfp (spec), pelB::lacO48 (kan), spoIID::cat, spoIIP::tet, spoIIIM::lox72,	1
bAT699	amyE::PspoIIQ-spoIVFRBS-lacI-gfp (spec), pbpG::lox72, pelB::lacO48 (kan), spoIID::cat, spoIIP::tet	1
bBD3	amyE::PspoIIQ-cfp(Bs) (cat), cprV::kan, spoIIIM::erm	This study
bBD17	amyE::PspoIIQ-cfp(Bs) (cat), spoIIIE:: lox72, ycgO::PspoIIIE-spoIIIE^{D584A}(phleo)	This study
bBD33	amyE::PspoIIQ-cfp(Bs) (cat), cprV::kan	This study
bBD34	amyE::PspoIIQ-cfp(Bs) (cat), cprV::kan, spoIIIE:: lox72, ycgO::PspoIIIE-spoIIIE^{D584A}(phleo)	
bBD35	amyE::PspoIIQ-cfp(Bs) (cat), cprV::kan, pbpG:: lox72	This study
bBD36	amyE::PspoIIQ-cfp(Bs) (cat), cprV::kan, pbpG:: lox72, spoIIIM:: lox72	This study
bBD64	amyE::PspoIIQ-cfp(Bs) (cat), cprV::kan, spoIIIM::erm, yhdG::PcprV-cprV(spec)	This study
bBD69	amyE::PspoIIQ-cfp(Bs) (cat), cprV::kan, yhdG::PcprV-cprV(spec)	This study
bBD126	amyE::PspoIIQ-cfp(Bs) (cat), cprV::kan, spoIIQ::erm	This study
bBD127	amyE::PspoIIQ-cfp(Bs) (cat), cprV::kan, spoIIIM::lox72, spoIIQ::erm	This study
bBD128	amyE::PspoIIQ-cfp(Bs) (cat), cprV::kan, pbpG::Markerless, spoIIQ::erm	This study
bBD129	cprV::kan, spoIIIE-gfp (spec)	This study
bBD130	cprV::kan, spoIIIE-gfp (spec), spoIIIM:: lox72	This study
bBD147	yhdG::PcprV-cprV-gfp(cat), cprV::kan	This study
bBD151	ycgO::PspoIIIE-spoIIIE^{D584A}(phleo), spoIIIE:: lox72, yhdG::PcprV-cprV-gfp(cat)	This study
bBD167	spoIIIE::neo, ycgO::spoIIIE36 (spec), yhdG::PcprV-cprV-gfp(cat)	This study
bBD169	amyE::PspoIIQ-cfp(Bs) (cat), cprV::kan, spoIID::spec, spoIIP::tet	This study
bBD170	amyE::PspoIIQ-cfp(Bs) (cat), cprV::kan, spoIID::spec, spoIIP::tet, spoIIIM:: lox72	This study
bBD171	amyE::PspoIIQ-cfp(Bs) (cat), cprV::kan, pbpG:: lox72, spoIIP::tet, spoIID::spec	This study
bBD172	amyE::PspoIIQ-cfp(Bs) (cat), cprV::kan, spoIID::spec, spoIIP::tet, spoIIQ::erm, spoIIIM:: lox72	This study
bBD173	amyE::PspoIIQ-cfp(Bs) (cat), cprV::kan, spoIID::spec, spoIIP::tet, spoIIQ::erm	This study
bBD174	amyE::PspoIIQ-cfp(Bs) (cat), cprV::kan, pbpG:: lox72, spoIIP::tet, spoIID::spec, spoIIQ::erm	This study
bBD188	amyE::PspoIIQ-spoIVFRBS-lacI-gfp (spec), cprV::kan, spoIIIM:: lox72, yycR::lacO48 (cat)	This study
bBD189	amyE::PspoIIQ-spoIVFRBS-lacI-gfp (spec), cprV::kan, yycR::lacO48 (cat)	This study
bBD189	amyE::PspoIIQ-spoIVFRBS-lacI-gfp (spec), cprV::kan, yycR::lacO48 (cat)	This study
bBD193	amyE::PspoIIQ-spoIVFRBS-lacI-gfp (spec), cprV:: lox72, pelB::lacO48 (kan)	This study
bBD196	amyE::PspoIIQ-spoIVFRBS-lacI-gfp (spec), cprV:: lox72, pelB::lacO48 (kan), spoIIIM::erm	This study
bBD197	amyE::PspoIIQ-cfp(Bs) (cat), cprV:: lox72, pbpG::kan, yhdG::PcprV-cprV(spec)	This study
bBD202	spoIIIM:: lox72, yhdG::PcprV-cprV-gfp(cat)	This study

bBD203	*pbpG::kan, yhdG::PcprV-cprV-gfp(cat)*	This study
bBD204	*pbpG::kan, spoIIIM:: lox72, yhdG::PcprV-cprV-gfp(cat)*	This study
bBD205	*spoIID::spec, spoIIP::tet, yhdG::PcprV-cprV-gfp(cat)*	This study
bBD207	*spoIIIE::kan, yhdG::PcprV-cprV-gfp(cat)*	This study
bBD210	*spoIID::spec, spoIIP::tet, spoIIIE::kan, yhdG::PcprV-cprV-gfp(cat)*	This study
bBD216	*amyE::PspoIIQ-spoIVFRBS-lacI-gfp (spec), cprV:: lox72, pelB::lacO48 (kan), spoIID::cat, spoIIP::tet*	This study
bBD221	*amyE::PspoIIQ-spoIVFRBS-lacI-gfp (spec), cprV:: lox72, pelB::lacO48 (kan), spoIID::cat, spoIIP::tet, spoIIIM::erm*	This study
bBD223	*amyE::PspoIIQ-cfp(Bs) (cat), cprV::kan, spoIIIE::phleo, ycgO::spoIIIEΔγ (spec)*	This study
bBD225	*spoIIIE::kan, ycgO::spoIIIEΔγ (spec), yhdG::PcprV-cprV-gfp(cat)*	This study
bBD235	*amyE::PspoIIQ-cfp(Bs) (cat), cprV::kan, pbpG::Markerless, spoIIP::tet, spoIID::spec, spoIIIM:: lox72*	This study
bBD227	*pbpG::kan, spoIIIE::phleo, spoIIIM:: lox72, yhdG::PcprV-cprV-gfp(cat)*	This study
bBD241	*amyE::PspoIIQ-spoIVFRBS-lacI-gfp (spec), cprV:: lox72, pbpG:: lox72, pelB::lacO48 (kan),spoIIIM::erm*	This study
bBD244	*amyE::PspoIIQ-spoIVFRBS-lacI-gfp (spec), cprV:: lox72, pbpG:: lox72, spoIIIM::erm, yycR::lacO48 (cat)*	This study
bBD245	*cprV::kan, pbpG:: lox72, spoIIIE-gfp (spec), spoIIIM:: lox72*	This study
bBD283	*amyE::PspoIIQ-spoIVFRBS-lacI-gfp (spec), cprV:: lox72, pbpG:: lox72, pelB::lacO48 (kan)*	This study
bBD284	*amyE::PspoIIQ-spoIVFRBS-lacI-gfp (spec), cprV:: lox72, pbpG:: lox72, yycR::lacO48 (cat)*	This study
bBD288	*amyE::PspoIIQ-spoIVFRBS-lacI-gfp (spec), cprV:: lox72, pbpG:: lox72, pelB::lacO48 (kan), spoIID::cat, spoIIP::tet*	This study
bBD289	*amyE::PspoIIQ-spoIVFRBS-lacI-gfp (spec), cprV:: lox72, pbpG:: lox72, pelB::lacO48 (kan), spoIID::cat, spoIIP::tet, spoIIIM::erm*	This study
bBD291	*amyE::PspoIIQ-cfp(Bs) (cat), cprV::kan, pbpG:: lox72, spoIID::spec, spoIIP::tet, spoIIIM:: lox72, spoIIQ::erm*	This study
bBD296	*amyE::PspoIIQ-cfp(Bs) (cat), cprV::kan, spoIIIM::erm, yhdG::PcprV-cprV (V4A)-His6 (spec)*	This study
bBD297	*amyE::PspoIIQ-cfp(Bs) (cat), cprV::kan, yhdG::PcprV-cprV (V4A)-His6 (spec)*	This study

Plasmids

**Name**	**Description**	**Source**
pBD06	*yhdG::PcprV-cprV (spec)*	This study
pBD07	*yhdG::PcprV-cprV-gfp (spec)*	This study
pBD14	*P_{lac}::T25-cprV (kan)*	This study
pBD16	*P_{lac}::T18-cprV (Amp)*	This study
pBD32	*P_{lac}::T18-cprV (V4A) (Amp)*	This study
pBD33	*P_{lac}::T18-cprV (N20A) (Amp)*	This study
pBD36	*P_{lac}::T18-cprV (V6A) (Amp)*	This study
pBD40	*yhdG::PcprV-cprV-His6 (spec)*	This study
pBD41	*yhdG::PcprV-cprV (V4A)-His6 (spec)*	This study
pBD42	*Plac::T18-cprV (V6A) (N20A) (amp)*	This study
pHC28	*P_{lac}::T18-spoIIIE (amp)*	*(Mohamed et al. 2022)*
pHC29	*P_{lac}::T18-spoIIIM (amp)*	*(Mohamed et al. 2022)*
pHC30	*P_{lac}::T18-pbpG (amp)*	*(Mohamed et al. 2022)*
pHC32	*P_{lac}::T25-spoIIIE (kan)*	*(Mohamed et al. 2022)*
pHC33	*P_{lac}::T25-spoIIIM (kan)*	*(Mohamed et al. 2022)*
pHC34	*P_{lac}::T25-pbpG (kan)*	*(Mohamed et al. 2022)*

Primers

**Name**	**Sequence**	**Source**
oBD27	ggaAAGCTTttatccggaaacgagataatcatc	This study
oBD28	gcgGGATCCttatttgcgatataaaataagggcgg	This study
oBD46	tgcCTCGAGtttgcgatataaaataagggcgg	This study
oBD68	cgcTCTAGAaatgctgaaggtagcggtatttgatg	This study
oBD69	cgcGGATCCcctgaaccagatttgcgatataaaataagggcgg	This study
oBD107	ggttcagggatgctgaaggcggcggtatttgatgaagag	This study
oBD108	ctcttcatcaaataccgccgccttcagcatccctgaacc	This study
oBD109	gacttacaaacggaaatcgcgtcatttttaaaagggatatc	This study
oBD110	gatatcccttttaaaaatgacgcgatttccgtttgtaagtc	This study
oBD114	ccattcgccagggggggatccttatttgcgatataaaataagggc	This study
oBD115	gagaaaggagagcggataaacatcaccaccaccatcac	This study
oBD116	tttatccgctctcctttctctatcaatctattc	This study
oBD117	ggatccccccctggcgaat	This study

*capital letters indicate restriction sites.

Plasmid Construction

pBD06 [*yhdG::PcprV-cprV (spec)*] was constructed by ligating a *Hind*III–*Bam*HI–digested PCR fragment containing the *cprV* region (amplified using primers oBD27 and oBD28 and 168 genomic DNA as template) into the corresponding *Hind*III–*Bam*HI sites of the pBB278 vector. pBB278 is an ectopic integration plasmid that mediates double cross-over insertion at the non-essential *yhdG* locus (B. Burton and D.Z.R., unpublished).

pBD07 [*yhdG::PcprV-cprV-gfp (spec)*] was constructed by ligating a *Hind*III–*Xho*I-digested PCR fragment containing the *cprV* DNA region (amplified using primers oBD27 and oBD46 and 168 genomic DNA as template), into the corresponding *Hind*III–*Xho*I sites of the pCR227 vector. pCR227 is an ectopic integration plasmid that mediates double cross-over insertion at the non-essential *yhdG* locus (Christopher Rodrigues, unpublished).

pBD14 [P_{lac}*::T25-cprV (kan)*] and **pBD16** [P_{lac}:: *T18-cprV (amp)*] were generated in a two-way ligation with *Xba*I-*Bam*HI PCR product containing the *cprV* gene [oligonucleotide primers oBD68 and oBD69 and 168 genomic DNA as template] and pKNT25[2] and pCH364[2], respectively, cut with *Xba*I and *Bam*HI.

pBD32 [P_{lac}*::T18-cprV (V4A) (amp)*] was constructed by site-directed mutagenesis of pBD16 with primers oBD107 and oBD108.

pBD33 [P_{lac}*::T18-cprV (N20A) (amp)*] was generated by site-directed mutagenesis of pBD16 with primers oBD109 and oBD110.

pBD36 [P_{lac}*::T18-cprV (V6A) (amp)*] was constructed by ligating an *Xba*I–*Bam*HI–digested DNA fragment containing the *cprV* open reading frame (including the V6A mutation)(provided by a GeneBlock; IDT), into the corresponding *Xba*I–*Bam*HI sites of pCH364.

pBD40 [*yhdG::PcprV-cprV-His6 (spec)*] was constructed by isothermal assembly of two PCR products, one containing *PcprV*–*cprV*–His6 including overhangs to the backbone of the pBB278 (*yhdG::spec*) (oligonucleotide primers oBD114 and oBD115 and 168 genomic DNA as template) and one containing the backbone pBB278 (oligonucleotide primers oBD116 and oBD117 using pBB278 as template).

pBD41 [*yhdG::PcprV-cprV (V4A)-His6 (spec)*] was constructed by ligating an *Hind*III–*Bam*HI–digested DNA fragment containing the *PcprV*–*cprV*–His6 open reading frame (including the V4A mutation) (provided by a GeneBlock; IDT), into the corresponding *Hind*III–*Bam*HI sites of pBB278.

pBD42 [*Plac::T18-crpV (V6A)(N20A) (Amp)*] was constructed by ligating an *Xba*I–*Bam*HI–digested DNA fragment containing the *cprV* open reading frame (including the V6A and N20A mutation) (provided by a GeneBlock; IDT), into the corresponding *Xba*I–*Bam*HI sites of pCH364 (*Plac::T18*)[2].

Supplementary References

1. Mohamed, A.M.T., Chan, H., Luhur, J., Bauda, E., Gallet, B., Morlot, C., Cole, L., Awad, M., Crawford, S., Lyras, D., et al. (2021). Chromosome Segregation and Peptidoglycan Remodeling Are Coordinated at a Highly Stabilized Septal Pore to Maintain Bacterial Spore Development. Dev Cell *56*, 36-51 e35. 10.1016/j.devcel.2020.12.006
2. Karimova, G., Pidoux, J., Ullmann, A., and Ladant, D. (1998). A bacterial two-hybrid system based on a reconstituted signal transduction pathway. Proc Natl Acad Sci U S A *95*, 5752-5756. 10.1073/pnas.95.10.5752.
