# Supplementary material for: A safeguarding factor monitors septal pore stability to secure compartmentalization during sporulation in Bacillus subtilis
Source: Commun Biol. 2026 Jul 15;9:1271. doi: 10.1038/s42003-026-10705-y (PMC13627676; doi:10.1038/s42003-026-10705-y)
Supplement: Supplementary file 4 [file 42003_2026_10705_MOESM3_ESM.docx]

**Description of Additional Supplementary Files**

**File name: Supplementary Data 1**

**Description: The numerical source data behind the graphs and Tn-seq data in the paper.**

**File name: Supplementary Video 1**

**Description: Video of CprV hexamer during computational simulation.**
